# Y225A induces long-range conformational changes in human prion protein that are protective in *Drosophila*

**DOI:** 10.1016/j.jbc.2023.104881

**Published:** 2023-06-02

**Authors:** Ryan R. Myers, Aliciarose John, Weiguanliu Zhang, Wen-Quan Zou, Alessandro Cembran, Pedro Fernandez-Funez

**Affiliations:** 1 Department of Biomedical Sciences, University of Minnesota Medical School, Duluth Campus, Duluth, Minnesota, USA; 2 Department of Chemistry and Biochemistry, University of Minnesota Duluth, Duluth, Minnesota, USA; 3 Department of Pathology and Neurology, National Prion Disease Pathology Surveillance Center, National Center for Regenerative Medicine, Case Western Reserve University, Cleveland, Ohio, USA

**Keywords:** prion protein, neurotoxicity, conformations, molecular simulations, Drosophila, protective mutations

## Abstract

Prion protein (PrP) misfolding is the key trigger in the devastating prion diseases. Yet the sequence and structural determinants of PrP conformation and toxicity are not known in detail. Here, we describe the impact of replacing Y225 in human PrP with A225 from rabbit PrP, an animal highly resistant to prion diseases. We first examined human PrP-Y225A by molecular dynamics simulations. We next introduced human PrP in *Drosophila* and compared the toxicity of human PrP-WT and Y225A in the eye and in brain neurons. Y225A stabilizes the β2-α2 loop into a 3_10_-helix from six different conformations identified in WT and lowers hydrophobic exposure. Transgenic flies expressing PrP-Y225A exhibit less toxicity in the eye and in brain neurons and less accumulation of insoluble PrP. Overall, we determined that Y225A lowers toxicity in *Drosophila* assays by promoting a structured loop conformation that increases the stability of the globular domain. These findings are significant because they shed light on the key role of distal α-helix 3 on the dynamics of the loop and the entire globular domain.

The prion protein (PrP) is a glycoprotein highly expressed in brain neurons that plays a central role in prion diseases, a heterogeneous class of neurodegenerative disorders in humans with direct molecular and pathological correlates in mammals (1, 2, 3, 4, 5). Sheep, goats, and cervids (all ruminants) are the only mammals known to develop endemic prion diseases (1). Several mammals proved susceptible to prion diseases in laboratory transmission experiments (chimpanzee, rodents) (2, 6, 7, 8), but not rabbits (9, 10). Other animals acquired prion diseases through zoonotic transmission during the mad-cow epidemic (bovine, felines, mustelids), but some seemed to escape despite being exposed: horses, pigs, dogs, and other canids (11, 12). Natural variation in the PrP sequence conferring different conformational dynamics of its globular domain is the likely mechanism underlying the different susceptibility of animals to prion diseases. Comparative studies reveal high structural conservation among mammals, consisting of three α-helices and a short β-sheet (13, 14, 15, 16, 17, 18). To date, it is unclear how subtle changes in natural PrP sequences impact its structure and toxicity, like the small differences identified in the key loop connecting β-sheet 2 and α-helix 2 (β2-α2 loop) and other subdomains (13, 14, 15, 16, 17, 19, 20, 21). Thus, identifying the key residues conferring conformational stability will contribute to uncover the molecular mechanisms mediating PrP neurotoxicity and disease susceptibility and new targets for therapeutics.

Rabbits are highly resistant to prion disease under experimental conditions that succeeded in transmission to rodents (9, 10). *In vitro* models demonstrate that cultured rabbit cells are a good environment to convert PrP from susceptible animals, indicating that the cellular milieu is not responsible for the high resistance to prion infections (22, 23). Moreover, specific fragments of rabbit PrP prevent conversion of chimeric rabbit-mouse PrP (24). Recent experiments showed that rabbits can replicate prions following multiple rounds of strain adaptation *in vitro*, but the rates of transmission are remarkably low after a second passage (25), supporting the idea that rabbits have remarkably low susceptibility to prion disease. We showed previously that hamster and mouse PrP expressed in *Drosophila* undergo progressive conformation changes and induce progressive brain degeneration and locomotor dysfunction (26). In contrast, rabbit PrP demonstrates high conformational stability and no toxicity in *Drosophila* (27, 28, 29). These results support the intrinsic conformational stability of rabbit PrP when expressed in a heterologous system. Ser174 (human PrP numbering throughout to avoid confusion, Fig. 1*A*) has been proposed to form a helix-capping domain that stabilizes α-helix 2 in rabbit PrP (30, 31). Recombinant rabbit PrP-S174N demonstrates lower resistance to denaturing agents than WT *in vit*ro, supporting a functional role for S174 (30). However, flies expressing rabbit PrP-S174N demonstrate no neurotoxicity (29). Thus, it is unclear whether (a) other residues are responsible for the low toxicity of rabbit PrP or (b) S174 combines with other rabbit-exclusive residues to stabilize its conformation. A recent survey identified 8 out of 11 changes between mouse and rabbit PrP (excluding the end of α-helix 3) as capable of increasing conversion of recombinant rabbit PrP *in vitro*, but S174 was negative in this study (32). Although several residues have been identified as potential mediators of the different properties of human and rabbit PrP, we still have a poor understanding of the structural correlates of PrP toxicity.

**Figure 1:**
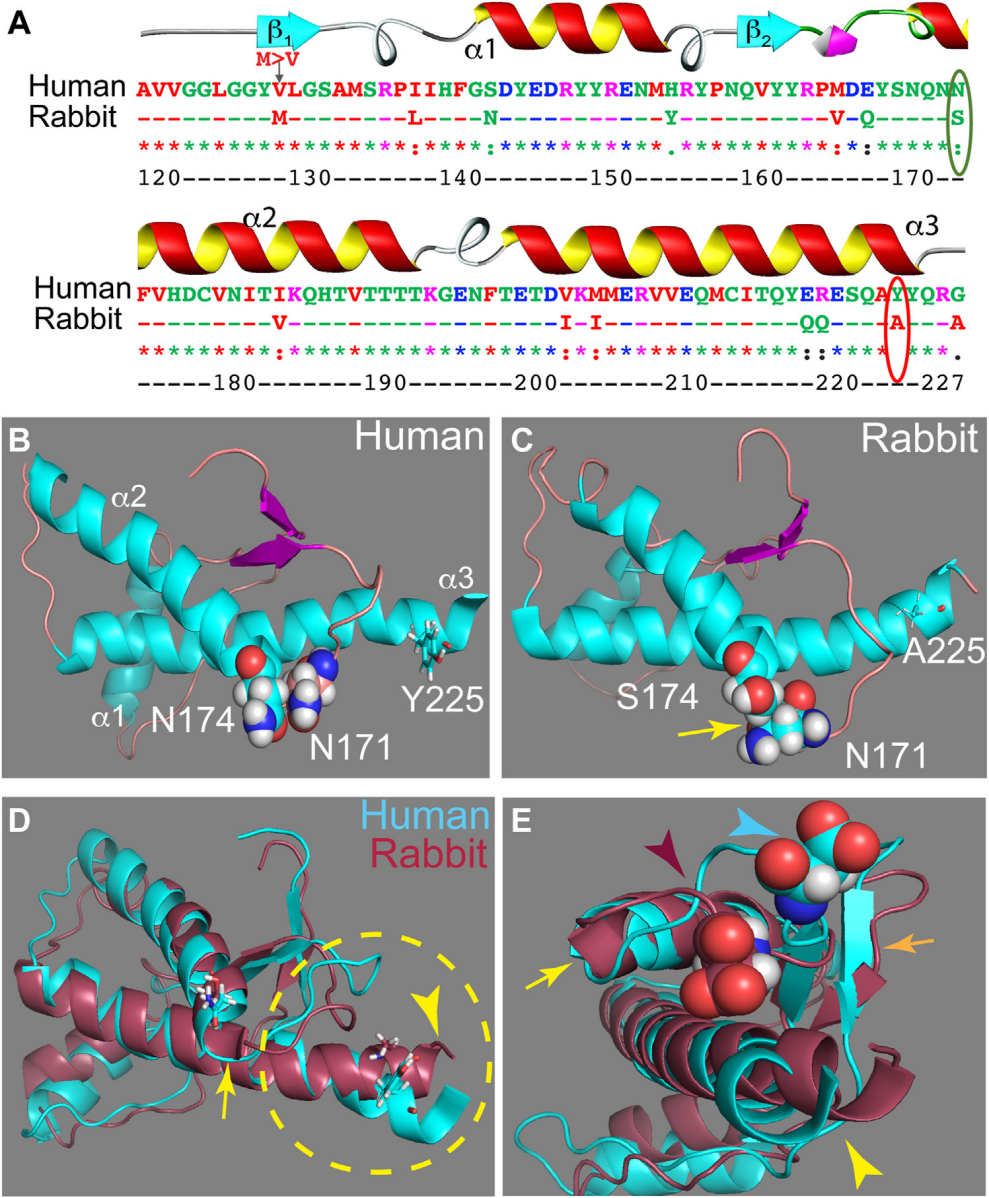
**The globular domain of human and rabbit PrP.***A*, alignment of the globular domain of human and rabbit PrP (amino acids 120–229, human PrP numbering). Two changes are *circled*: N/S174 and Y/A225. *B*–*E*, structures of the globular domain of human (1QM2) and rabbit (2FJ3) PrP. N/S174 and Y/A225 are represented as *sticks* or *spheres*. The interaction of N171 with S174 is shown (*C*, *arrow*). *D* and *E*, 3D alignment of the globular domains of human and rabbit PrP. The 3D domain comprised of the β2-α2 loop and helix 3, the CT3DD, is *circled*. *Arrows* point to differences. CT3DD, C-terminal 3D domain; PrP, Prion protein.

Human and rabbit PrP contain several amino acid differences in the β2-α2 loop and distal α-helix 3. Interestingly, these subdomains form a 3D epitope, which we termed the “C-terminal 3D domain (CT3DD)”, proposed to be critical for the initiation of PrP misfolding (33, 34). Modifications in the sequence of the CT3DD domain suggest a potential to modulate PrP conformational dynamics (35, 36). Here, we focused on Y/A225, which has received little attention despite its potential interactions with the loop. Molecular dynamics (MD) simulations revealed robust effects of Y225A on the β2-α2 loop and overall stabilization of the CT3DD in a 3_10_-helix compared to a more dynamic PrP-WT that explores at least six different conformations. We generated transgenic flies expressing human PrP-WT and Y225A and found that Y225A significantly suppress PrP-WT toxicity in several neurotoxicity assays. Y225A also accumulates lower levels of Na+ phosphotungstic acid (NaPTA)-insoluble conformations, supporting the *in silico* predictions and the reduced toxicity *in vivo*. In conclusion, Y225A contributes to the conformational stability of the CT3DD, resulting in lower toxicity.

## Results

### Sequence and conformational differences between human and rabbit PrP

To begin deciphering the determinants of PrP toxicity, we first conducted sequence and structural alignments of the globular domain of human and rabbit PrP. The sequence alignment reveals high conservation, with 14 amino acid changes, six of which are nonconservative (Fig. 1*A*). The structure of the globular domains reveals modest differences on a larger scale (Fig. 1, *B*–*E*) (30, 31, 37), but when focusing on the CT3DD, rabbit PrP shows a shorter β-sheet, longer α-helix 2, and—importantly—closer contacts between the β2-α2 loop and α-helix 3 (Fig. 1, *B*–*E*). Some amino acid differences in the β2-α2 loop are shared between rabbit and rodent PrP and, thus, are poor candidates to mediate the increased stability of rabbit PrP. S174 (Fig. 1*A*, circled) is proposed to form a helix-capping domain with N171 that stabilizes α-helix 2 (Fig. 1, *B* and *C*) (30). However, rabbit PrP-S174N failed to increase recombinant conversion *in vitro* (32) and to increase toxicity in flies (29, 30, 31), suggesting a modest impact. The C-terminal portion of α-helix 3 has a significant change: Y225 in human and A225 in rabbit (Fig. 1, *A*–*C*). Y/A225 are different in the bulk of the side chains, while the hydroxyl group on Tyr provides added functionality as a hydrogen bond donor and acceptor. We hypothesize that Y/A225 contribute to the structural differences between rabbit and human PrP. To test this idea, we examined the consequences of introducing Y225A on the human PrP backbone by two complementary strategies: MD simulations and *in vivo* studies in transgenic *Drosophila*.

### Y225A alters the conformational dynamics of the β2-α2 loop in human PrP

**Figure 2:**
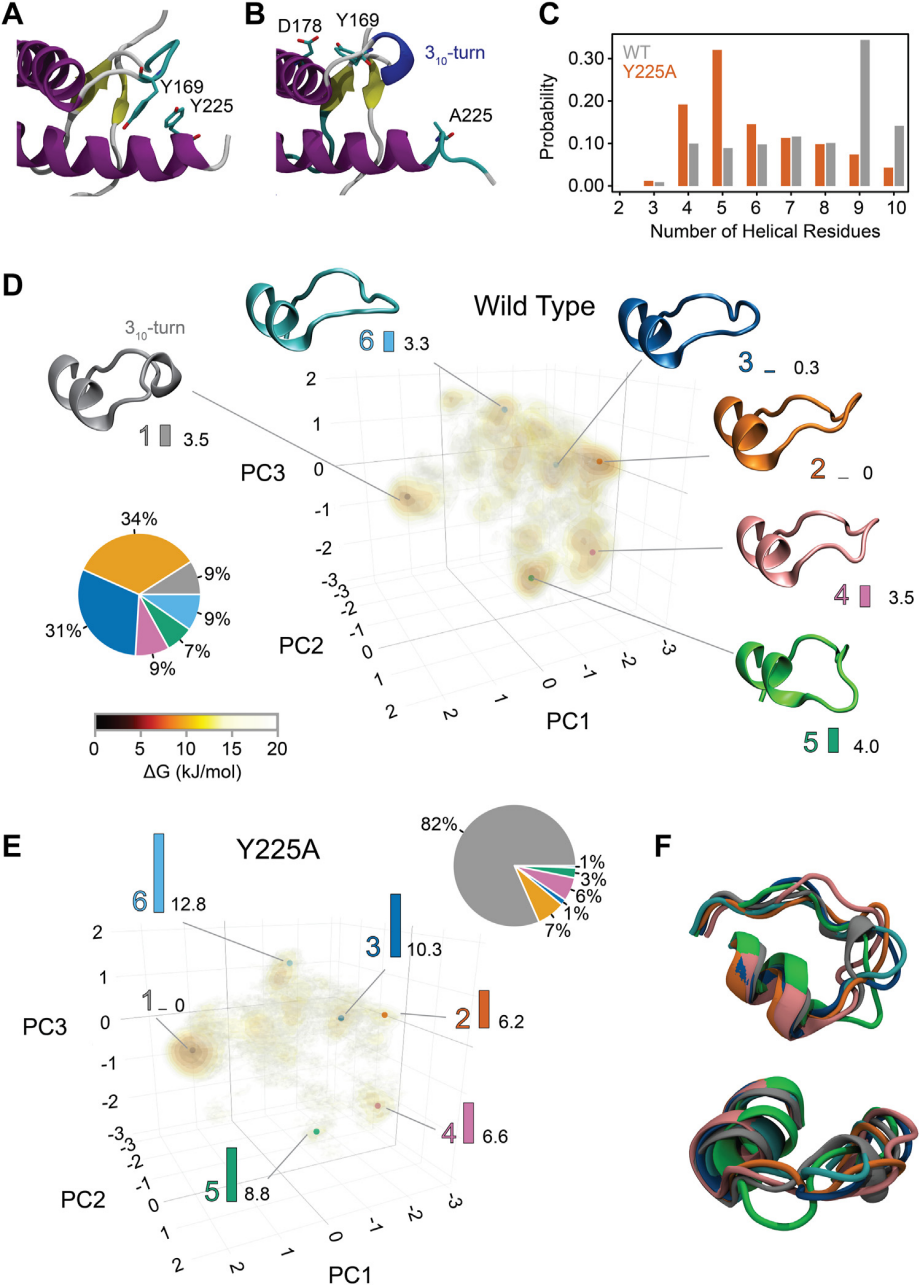
**Structure of PrP-WT and Y225A.***A*, representative structure of the CT3DD in PrP-WT with the Y225–Y169 interaction. *B*, representative structure of Y225A, with the Y169–D178 interaction. *C*, α-helix content of the distal α-helix 3 for WT (*gray*) and Y225A (*orange*). *D* and *E*, Gibbs energy surface for PrP-WT and Y225A. The axes correspond to the first three eigenvectors of the dihedral-space PCA. Seven Gibbs energy isocontours were drawn between 0 and 25 kJ/mol according to the color bar, which identify six clusters (color-coded). The relative Gibbs energy of each cluster with respect to the lowest energy (cluster 2 in WT, cluster 1 in Y225A) is plotted as *vertical bars* with the value of the ΔG in kJ/mol reported to the *right of the bar*. A representative structure for each cluster is shown. The cluster population is reported as pie charts using the same color-coding, with percentages indicating the population of each cluster. *F*, side and top view of the 3D alignment of the representative structures from each cluster shown in (*D*). CT3DD, C-terminal 3D domain; PCA, principal component analysis; PrP, Prion protein.

To sample the accessible conformational space, we performed temperature replica exchange MD simulations (T-REMD). The β2-α2 loop shows the largest difference between human PrP-WT and Y225A trajectories. In WT, the β2-α2 loop appears quite dynamic and mostly adopts an extended conformation over α-helix 3 (Fig. 2*A*). In Y225A, the loop is relatively static and samples preferentially a 3_10_-helix for M166-E168 (Fig. 2*B*). In addition, Y225A has a lower helical content in the C-terminus region of the α-helix 3 (Fig. 2*C*). Since the backbone dihedral φ/ψ angles define the secondary structure, we performed principal component analysis (PCA) on the φ/ψ angles of the loop. We used the 48 sines and cosines from the φ and ψ dihedral angles from R164 to F175 and identified the linear combinations (*eigenvectors*) that maximize the variance. When the data is projected on these eigenvectors, the first three capture 55% of the information (Fig. S1*A*), in line with similar analyses (38). The major contributions derive from residues 166 to 171 (Fig. S1*B*), which form the central section of the loop. The sine/cosine data projections along the first three eigenvectors are shown as Gibbs energy isodensity plots for human PrP-WT and Y225A (Fig. 2, *D* and *E*). For WT, the six most populated clusters are identified as low Gibbs energy regions (Fig. 2*D*). The center for each cluster and the relative Gibbs energy with respect to the lowest energy (cluster 2) are displayed (Fig. 2*D*). The alignment for R164-F175 highlights the six different conformations adopted by the β2-α2 loop (Fig. 2*F*). The Gibbs free energy difference between conformations is small, with the most and least stable differing only by 4.0 kJ/mol. The pie chart in Figure 2*D* shows that these conformations have similar populations in the 10 to 30% range. These clusters are connected through low energy barriers (20 kJ/mol or lower), which correspond to transition times in the nanosecond scale. In contrast, PrP-Y225A dominantly populates cluster 1 (82%), which is characterized by a 3_10_-helix conformation (Fig. 2*E*). The other conformations have only marginal populations (Fig. 2*E*) and are accessible through energy barriers higher than in the WT.

**Figure 3:**
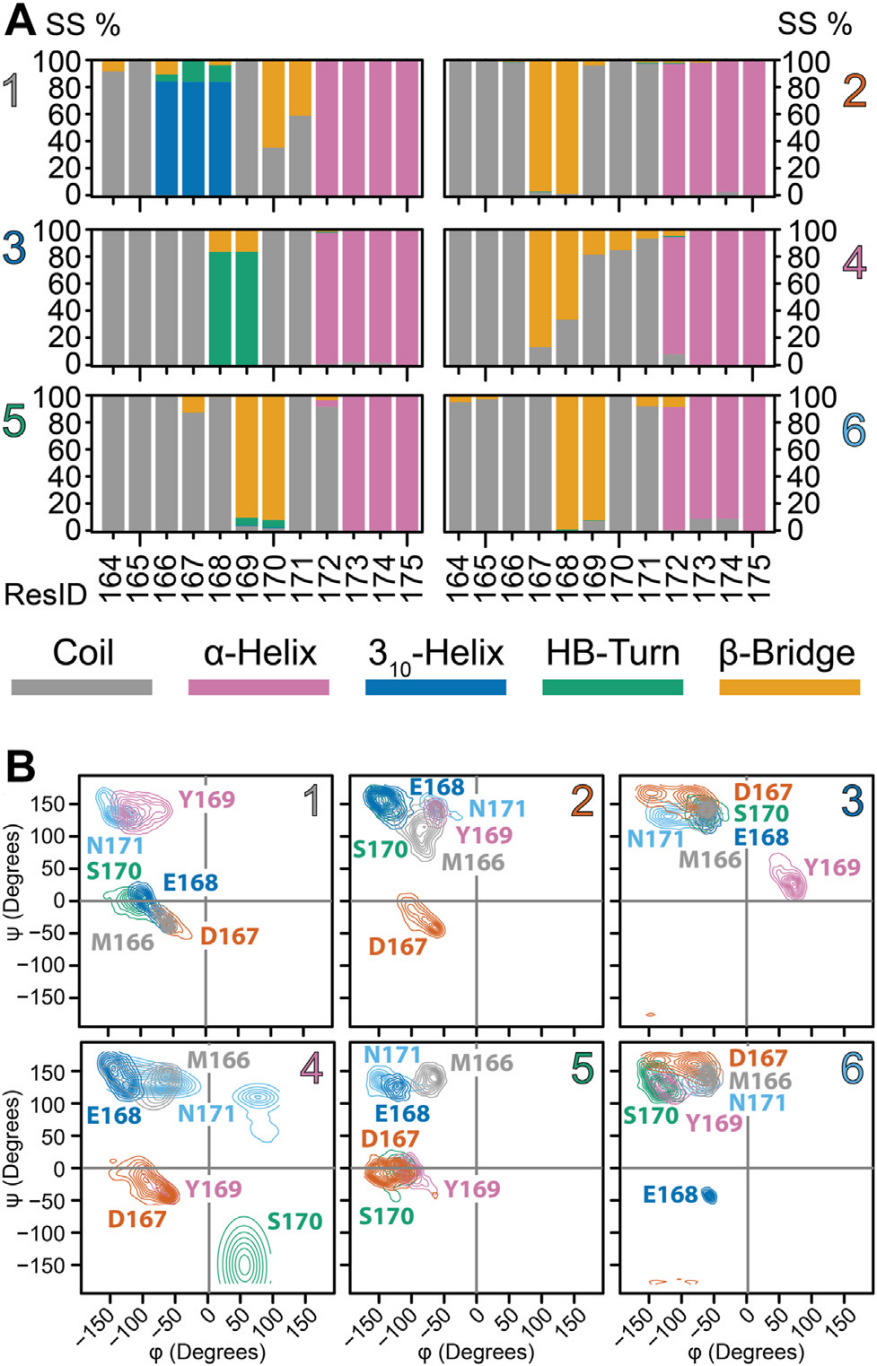
**Secondary structure of WT and Y225A.***A*, DSSP analysis of the structure of β2-α2 loop residues for each cluster. For every residue in the loop, the percent occupancy of each DSSP assignment is color coded. *b*, Ramachandran plots for residues 166 to 171 for each cluster are plotted as isocontours along the φ and ψ angles. Each angle is identified by a different color in each panel. DSSP, dictionary of protein secondary structure.

To characterize the clusters, we used the dictionary of protein secondary structure (DSSP) (39) (Fig. 3*A*). DSSP confirmed the validity of the PCA clustering, as each cluster has its unique fingerprint. In cluster 1, M166–E168 adopt a 3_10_-helix, α-helix 2 starts at Q172, and residues S170 and N171 form a bend (39) (Fig. 3*A*). Clusters 2, 4, 5, and 6 are characterized by different bends, whereas cluster 3 forms a hydrogen-bonded turn involving E168 and Y169 (Fig. 3*A*). Next, we examined the dihedral angles in the β2-α2 loop as Ramachandran plots (Fig. 3*B*). In cluster 1, residues 166 to 168 occupy the region characteristic to the 3_10_-helix (Fig. 3*B*). To further distinguish the secondary structure within the loop, we used PROMOTIF (40), which classifies the secondary structure based on backbone dihedral angles and distances between atoms (Table 1). 166 to 168 form a 3_10_-helix in cluster 1 in 80% of instances, whereas the high curvature regions for all other clusters were classified as β-turns—clusters 2, 4, and 6 as type VIII and cluster 3 as type II. Type IV is a catch-all for unmatched turns, like cluster 5.

**Table 1:** Secondary structure assignments for clusters 1 to 6

Cluster	i	i + 1	i + 2	i + 3	PROMOTIF assignment	%
1	M166	D167	E168	NA	3_10_-helix	82%
2	M166	D167	E168	Y169	β-turn (IV/VIII)	44%/40%
3	D167	E168	Y169	S170	β-turn (II/IV)	62%/35%
4	M166	D167	E168	Y169	β-turn (VIII/IV)	34%/34%
5	E168	Y169	S170	N171	β-turn ((IV_4_)^a^/I)	71%/21%
6	D167	E168	Y169	S170	β-turn (VIII)	90

For each cluster, the residues involved in the turn are reported together with the % for each PROMOTIF assignment.

a Type IV_4_ was identified by comparing the dihedral angles’ Ramachandran space with a previous report (99).

### Y225A favors the 3_10_-helix conformation by destabilizing the β-turns

**Figure 4:**
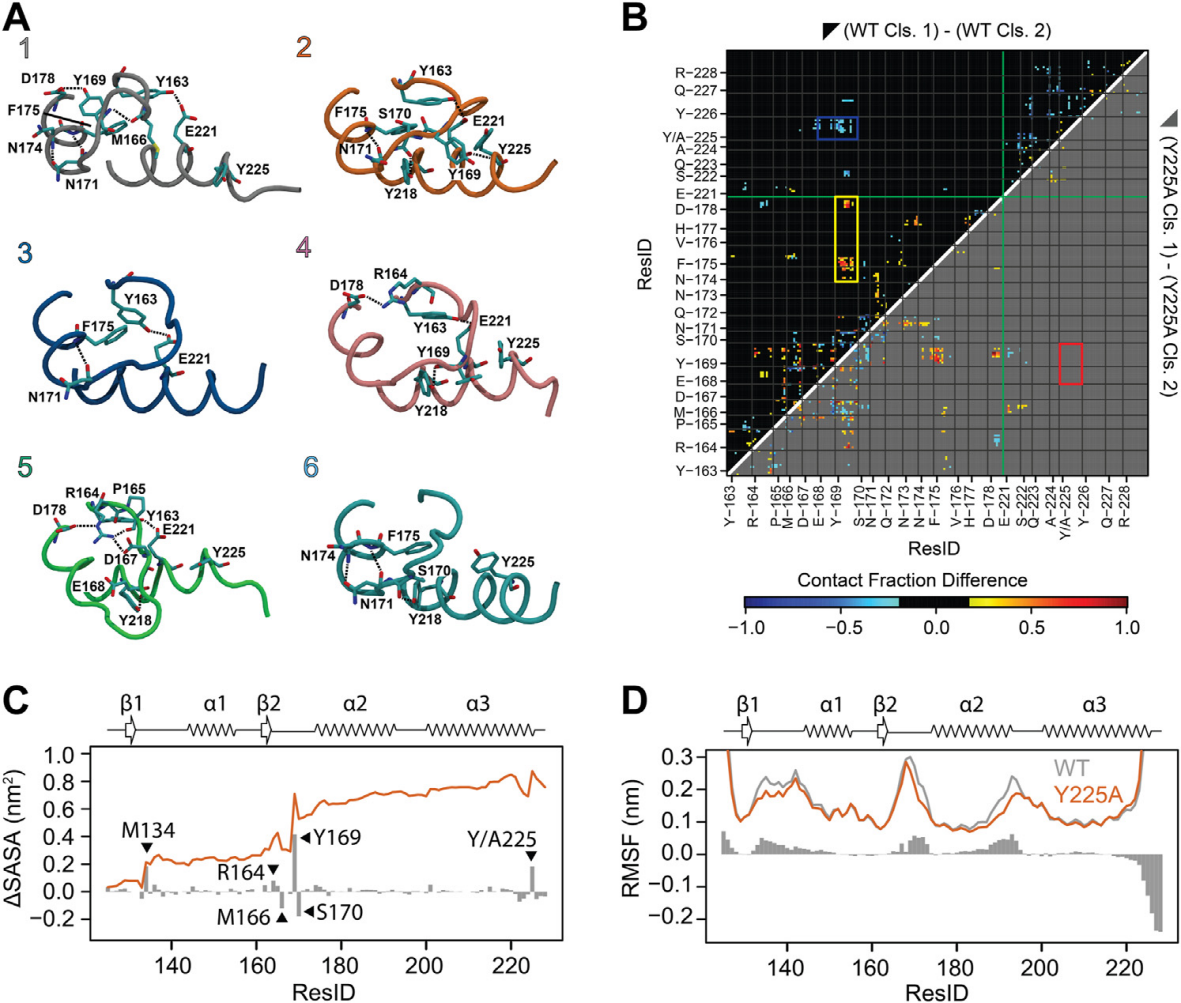
**Hydrogen bond networks and contact maps in WT and Y225A.***A*, hydrogen bond networks for PrP-WT clusters: showing differences (*arrows*) and over 50% occupancy. *B*, contact maps difference between cluster 2 and cluster 1 for WT (*top*, *black*) and Y225A (*bottom*, *gray*). Only the residues 163 to 178 and 221 to 228 are reported for clarity. Differences in contacts below 20% were ignored. A *red box* identifies the missing interactions in Y225A (168-169/225). *C*, solvent accessible surface area (SASA). *Vertical bars* show the difference in the hydrophobic SASA between WT and Y225A. A positive value means WT has a larger hydrophobic SASA than Y225A. Residues showing large changes are labeled. *Orange line*, cumulative difference in hydrophobic area: 0.9 nm^2^. *D*, root mean squared fluctuations (RMSFs) for WT (*gray*) and Y225A (*orange*). Bars show the difference in RMSF: a positive value indicates increased dynamics in WT. PrP, Prion protein.

We next analyzed the hydrogen bond pattern in each cluster (Table S1). The 3_10_-helix is stabilized by a hydrogen bond between the side chains of Y169 and D178 (41, 42, 43) (Fig. 4*A*). This hydrogen bond is not present in any of the β-turns, in which D178 and R164 side chains form an ionic bond (Fig. 4*A* and Table 1). The only other cluster in which Y169 is involved is cluster 4, where its main chain accepts a hydrogen bond from the side chain of Y218 (Fig. 4*A*). Cluster 5 is stabilized through a network where the side chain of R164 is hydrogen bonded with the D167 and D178 side chains and to the P165 carbonyl (Fig. 4*A*). Cluster 2 is the only one where Y225 forms a backbone hydrogen bond with E221 as part of α-helix 3 (51%) (Fig. 4*A*). Although previous work has shown that the β-turns in mouse PrP are stabilized by hydrogen bonds between S170 and D167 (42), we did not find evidence for this in human PrP. We attribute this difference to the residue at 166 (M166 in human; V166 in mouse), which is involved in steric crowding in the loop region.

We next mapped the differences in contacts between clusters 1 and 2 in PrP-WT and Y225A. In PrP-WT cluster 1, Y169 makes more interactions with N174, F175, and D178 (Fig. 4*B*, yellow box), consistent with the hydrogen bond between Y169 and D178 and the π-stacking of Y169 and F175 (41, 42, 43). In WT cluster 2, Y225 makes more interactions with Y169 and E168 (Fig. 4*B*, blue box). This is unexpected from the hydrogen bond analysis, which led us to conclude that the interaction between the two side chains is due to π-stacking between the two aromatic side chains. In the Y225A contact map (Fig. 4*B*, gray), the π-stacking between A225 and Y169 is not possible, and there are no differences between clusters 1 and 2 in that region (Fig. 4*B*, red box). Thus, Y225A destabilizes the β-turn by eliminating the Y225/Y169 π-stacking, thus shifting the population to the 3_10_-helix.

### Y225A decreases the hydrophobicity and dynamics in the β2-α2 loop

Next, we analyzed the exposure of hydrophobic surfaces to the solvent in PrP-WT and Y225A by mapping the difference in the hydrophobic solvent accessible surface area (SASA). Due to the larger side chain, Y225 in WT has a larger value than A225 in Y225A (Fig. 4*C*). Yet, the largest impact is in the β2-α2 loop, where Y169 is significantly more exposed to the solvent in WT than in Y225A (Fig. 4*C*). The cumulative hydrophobic SASA (Fig. 4*C*, orange line) shows that WT exposes about 1 nm^2^ more of hydrophobic SASA than Y225A, of which about half is due to Y169. M134, which is at the end of the β1 strand, is also more exposed to solvent in the WT because of increased dynamics (see RMSF in Fig. 4*D*). In contrast, in Y225A, M134 is neatly packed between M213 and R220 side chains, suggesting that Y225A impacts regions that are not in direct contact with it. The changes in the dynamics of PrP upon mutation are shown as the root mean squared fluctuations (RMSFs) for every residue (Fig. 4*D*). Gray and orange lines display the absolute RMSF, whereas the gray histogram reports the difference between WT and Y225A. Overall, WT and Y225A have similar fluctuation patterns, but the WT shows increased dynamics in the β2-α2 loop as well as in the terminal part of the β1 strand and of the α2 helix. The last few residues of α-helix 3 are more mobile in Y225A due to its lower helical content.

### N174S and Y225A partially suppress the toxicity of human PrP in flies

**Figure 5:**
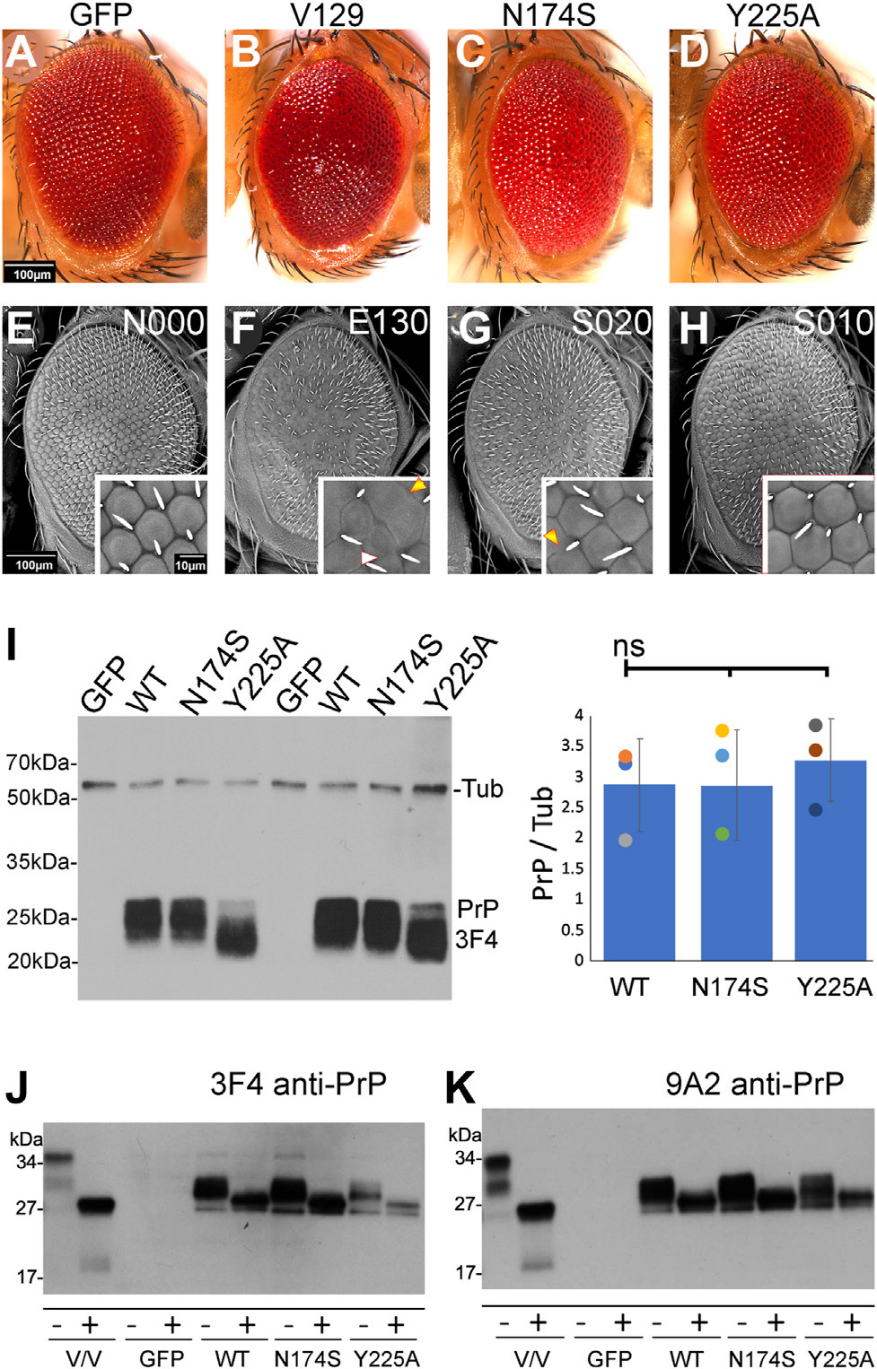
**N174S and Y225A suppress the toxicity of human PrP-WT in the eye.** Micrographs of eyes from frozen flies (*A*–*D*) or fixed for the scanning electron microscope (*E*–*H*) from control flies expressing mCD8-GFP or human PrP in the eyes (*GMR-Gal4*). Control flies (*UAS-mCD8-GFP-attP2*) show highly organized eyes with hexagonal ommatidia (*A* and *E*, *inset*). Flies expressing PrP-WT (*UAS-human PrP-V129-attP2*) show small and disorganized eyes (*B*) with fused ommatidia and irregularly spaced bristles (*F*, *inset*). Flies expressing N174S (*UAS-human PrP-N174S*-*attP2*) show mildly disorganized eyes (*C* and *G*, *arrowheads*). Flies expressing Y225A (*UAS-human PrP-Y225A*-*attP2*) show almost normal eyes (*D* and *H*, *inset*). Scores: 0 (no change) to 3 (largest change) in size (S), organization (O), and pigmentation (P). *I*, Western blot from fly extracts expressing GFP or PrP in the fly eye probed with anti-β-Tubulin and 3F4 anti-PrP antibodies in two biological replicates. Quantification of total PrP shows no significant differences (pairwise *t* test): V129 *versus* N174S, *p* = 0.76; V129 *versus* Y225A, *p* = 0.538; Y225A *versus* N174S, *p* = 0.79. *J* and *K*, deglycosylation assays. Western blot of PrP treated with (+) or without (−) PNGase F probed with 3F4 (*J*) or 9A2 (*K*) anti-PrP antibodies. The first two lanes show human brain homogenate from homozygous PrP-V129 (VV). PNGase F, peptide N-glycosidase F; PrP, Prion protein.

Following the results from the MD simulations, we generated transgenic flies carrying human PrP-WT and Y225A in a human V129 backbone inserted in the same locus, attP2. We also created flies expressing the better characterized N174S substitution as reference in the same background (28, 30, 44). This fly model expressing highly toxic human PrP (45, 46) provides a sensitive model to examine the impact of candidate protective mutations under comparable expression levels in fly neurotoxicity assays. We first expressed human PrP in the eye and examined the eye phenotype as a preliminary, easily accessible assay. We created a subjective descriptive score for the phenotypes of fly eyes focusing on three categories, size, organization, and pigmentation, each scored 0 (no effect) to 3 (robust change) and a summative descriptor (No effect (N), Enhancer (E), Suppressor (S)). Compared to control flies expressing GFP (N0/0/0, Fig. 5, *A* and *E*), expression of PrP-WT results in slightly smaller eyes and poorly differentiated ommatidia or “glassy” eyes (E1/3/0) (Fig. 5, *B* and *F*) (46). Flies expressing N174S exhibit larger yet still disorganized eyes (S0/2/0) (Fig. 5, *C* and *G*). Flies expressing Y225A show highly organized eyes, with well-defined and regular ommatidia (S0/1/0) (Fig. 5, *D* and *H*). Quantification of these cumulative eye scores showed significant differences among all genotypes (*p* < 10^−4^, Table S2) Overall, these preliminary observations support the lower toxicity of N174S and Y225A than PrP-WT, with Y225A showing a more significant protective effect.

### N174S and Y225A biogenesis is similar to PrP-WT

We homogenized heads from flies expressing GFP or PrP in the eye and detected the electrophoretic mobility of PrP by Western blot. The total amount of PrP expressed from each construct is similar as expected from the shared insertion locus (Fig. 5*I*). PrP has two facultative *N*-glycosylation sites, resulting in three glycoforms. WT and N174S exhibit identical electrophoretic patterns (Fig. 5*I*), whereas Y225A runs at a slightly lower size (Fig. 5*I*). Y225A appears to undergo different glycosylation, with a weak diglycosylated band and a more prominent unglycosylated band compared with WT and N174S. We next performed complete deglycosylation assays using peptide N-glycosidase F (PNGase F) to examine if the differences in mobility are due to glycosylation or possible alterations in PrP biogenesis. Control PrP from human brain homogenates shows a diglycosylated band running at a higher molecular weight than *Drosophila*-expressed human PrP due to the smaller glycans in insects than mammals (Fig. 5, *J* and *K*, lane 1) (47, 48). Deglycosylation of human PrP yields one band at 25 kDa consistent with full-length unglycosylated PrP and a band at 20 kDa corresponding to the C2 proteolytic fragment (Fig. 5, *J* and *K*, lane 2). We obtained similar results when PrP is detected with different antibodies, 3F4 and 9A2 (Fig. 5, *J* and *K*). Deglycosylation of PrP-WT, N174S, or Y225A expressed in *Drosophila* produced a similar band for all constructs around 25 kDa visualized with 3F4 and 9A2, suggesting that the biogenesis of Y225A is comparable to WT in *Drosophila*. The slight differences in Y225A mobility observed in Figure 5, *I*–*K* can be explained by the different handling of homogenates for these two procedures, resulting in all variants running at the expected 25 kDa band for full-length, unglycosylated PrP following PNGase treatment (Fig. 5, *F* and *K*).

**Figure 6:**
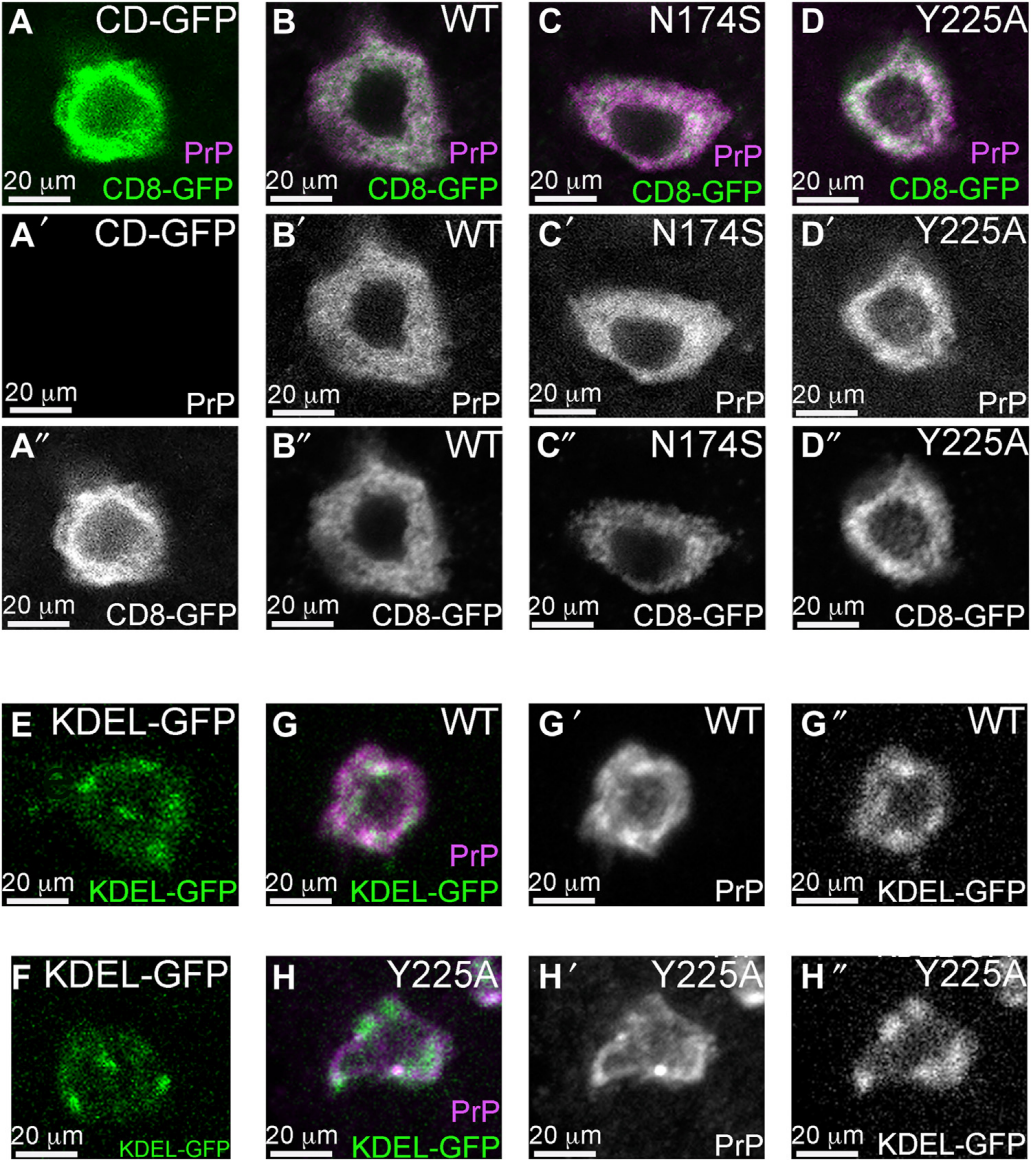
**Subcellular distribution of human PrP in *Drosophila* interneurons.***A*–*A"*, expression of CD8-GFP alone in the larval brain (*OK107-Gal4; UAS-CD8-GFP*) highlights the ER, secretory pathway, and membrane distribution of CD8-GFP. *B*–*D"*, co-expression of CD8-GFP and human PrP-WT, N174S, or Y225A in the same interneurons resulted in similar diffuse distribution as CD8-GFP and compared to each other. *E* and *F*, expression of GFP-KDEL alone in the same interneurons shows accumulation in a few puncta that identify the ER–Golgi complexes. *G*–*H"*, co-expression of GFP-KDEL and human PrP-WT or Y225A in the same interneurons shows partial colocalization in the ER-Golgi and additional accumulation of PrP in secretory compartments and membranes. Larval brains were fixed and permeabilized. PrP, Prion protein.

**Figure 7:**
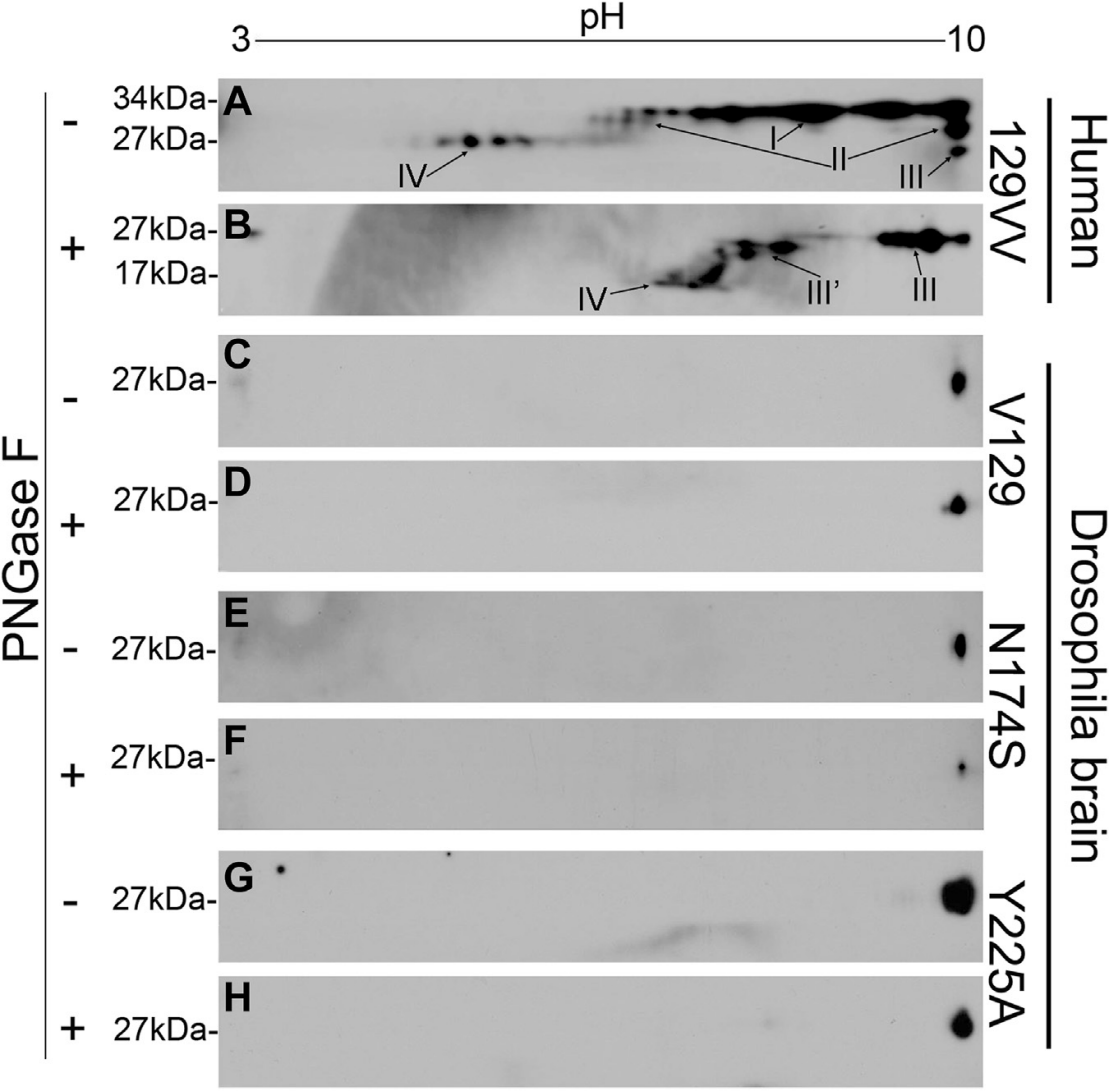
**2D blot analysis of PrP from human brain and *Drosophila*.** 2D blotting of PrP from human brain and *Drosophila* homogenates with or without PNGase F treatment. *A* and *B*, human brain PrP-129VV; *C* and *D*, *Drosophila* expressing PrP-WT (*GMR-Gal4/UAS-human PrP-V129-attP2*). *E* and *F*, PrP-*Drosophila* expressing N174S (*GMR-Gal4/UAS-human PrP-N174S*-*attP2*); g and h: *Drosophila* expressing PrP-Y225A (*GMR-Gal4/UAS-human PrP-V129-Y225A*-*attP2*). *A*, *C*, *E*, and *G*, homogenates not treated with PNGase F. *B*, *D*, *F*, and *H*, homogenates treated with PNGase F. Membranes were probed with the 3F4 anti-PrP antibody. PNGase F, peptide N-glycosidase F; PrP, Prion protein.

To gain additional insight about the biogenesis of WT and mutant PrP in fly cells, we examined its subcellular distribution in brain neurons as an indicator of posttranslational modification and maturation through the secretory pathway. We recently characterized the subcellular distribution of human, mouse, and hamster PrP expressed in fly neurons showing the human PrP is present in different compartments of the secretory pathway (46). Here, we co-expressed WT, N174S, and Y225A with chimeric CD8-GFP, which shows robust membrane accumulation and we used for labeling cell bodies, axonal projections, and dendritic fields of mushroom body neurons in the adult brain (Figs. 6 and 7) (49). In fixed and permeabilized larval brains, CD8-GFP shows both membrane and intracellular accumulation in interneurons expressing OK107-Gal4 (Fig. 6*A*). PrP-WT, N174S, or Y225A show extensive colocalization with CD8-GFP (Fig. 6, *B*–*D*) corresponding to several secretory compartments, as we showed recently (46). To examine whether any fraction of PrP is retained in the ER-Golgi, we examined the distribution of KDEL-GFP which labels the ER and proximal Golgi. Expression of GFP-KDEL alone shows a punctate distribution in larval brain interneurons (Fig. 6, *E* and *F*). Co-expression of GFP-KDEL and PrP-WT or Y225A did not alter KDEL-GFP punctate distribution (Fig. 6, *G* and *H*). Additionally, GFP-KDEL showed partial colocalization with PrP (Fig. 6, *G* and *H*), indicating that PrP is processed through the ER-Golgi but is not retained there. Thus, WT, N174S, and Y225A show similar subcellular distribution in the secretory pathway indicating that N174S and Y225A do not introduce overt alterations in the biogenesis of human PrP. Along with the electrophoretic mobility described above, human PrP appears to undergo full posttranslational modification, which requires exit from the Golgi complex.

### Glycosylation of human PrP expressed in *Drosophila*

Posttranslational glycosylation and sialylation add weight and negative charge to PrP (50, 51). The mammalian PrP glycopattern can be highly complex because the number and type of sugar residues can vary significantly. 2D-PAGE examination of PrP from human brain homogenate shows multiple spots that represent the complexity of the monoglycosylated and diglycosylated isoforms (Fig. 7). Consistent with our previous observation (52), human brain exhibits PrP spots I, II, III, and IV corresponding to di-, mono-, and un-glycosylated full-length PrP as well as N-terminally truncated diglycosylated PrP (Fig. 7*B*). After PNGase F treatment, only deglycosylated full-length (III) and truncated PrP spots (III’ and VI) are detected. The shifts confirm that all PrP spots except III are glycosylated species. In contrast, human PrP-WT, N174S, or Y225A expressed in *Drosophila* migrated to similar positions at approximately *p*I 10 and 28 to 29 kDa (Fig. 7, *C*, *E* and *G*). After deglycosylation, the PrP spot migrated slightly faster but still only one spot is detected in each variant (Fig. 7, *D*, *F* and *H*), confirming that PrP is significantly less glycosylated in *Drosophila*. Coomassie-R blue confirms that the *Drosophila* homogenates are well separated (Figs. 7*C* and S2). Overall, the high complexity of human PrP is not observed in PrP expressed in flies possibly due to lacking the variable sialylation steps added to mature PrP. Since PrP-WT and Y225A show similar glycosylation profiles in flies, this does not contribute to their different toxicity.

### N174S and Y225A are protective against the progressive degeneration of brain neurons

**Figure 8:**
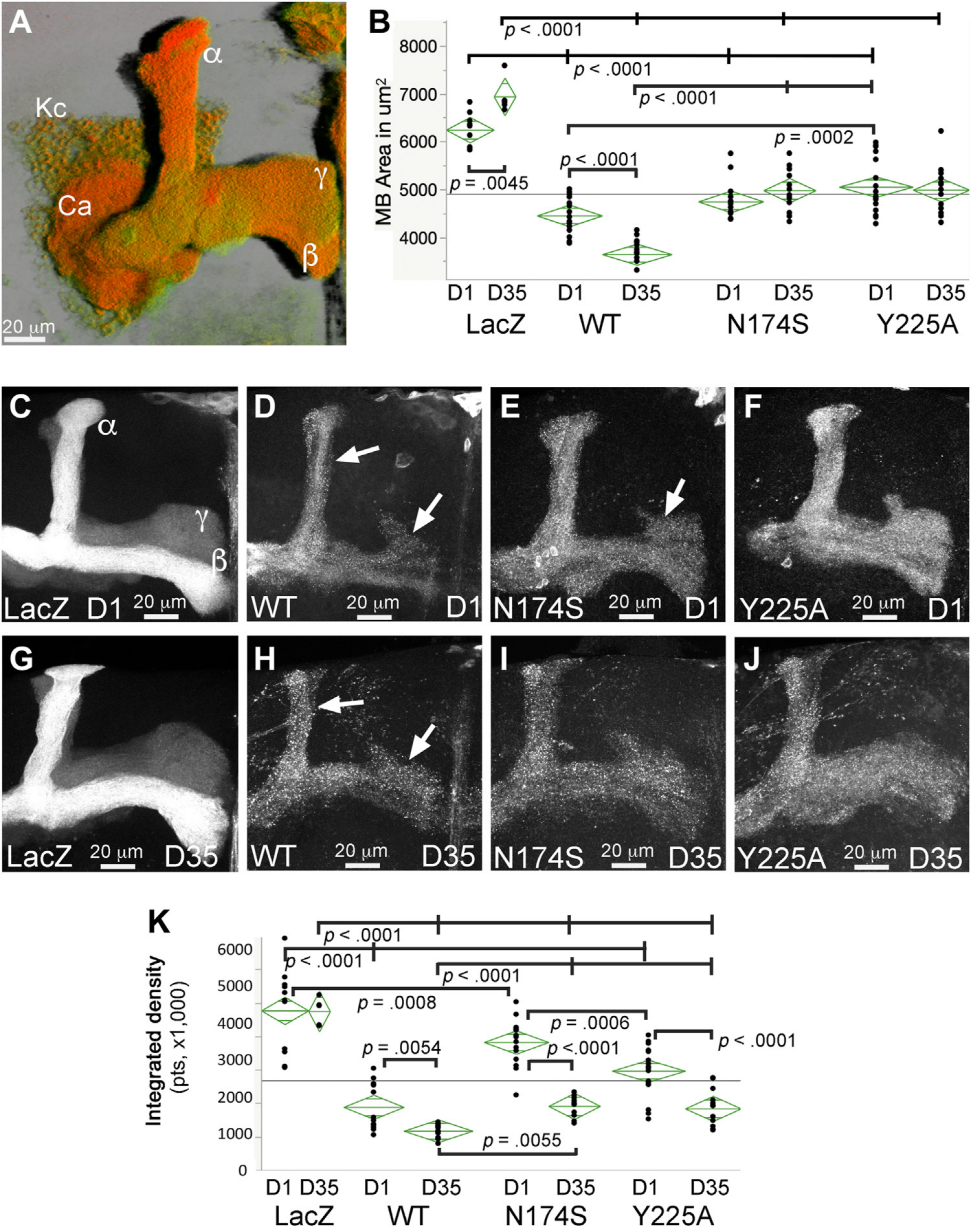
**N174S and Y225A lower the toxicity of human PrP in mushroom body axonal projections.***A*, 3D rendering of the *Drosophila* mushroom body complex. The cell bodies of the Kenyon cells (Kc) are in the posterior brain, and the dendritic fields, the calix (Ca), lie beneath the cell bodies. The axonal projections travel to the front of the brain and split into dorsal (α) and medial (β and γ) branches. *B*–*K*, imaging and quantification of axonal projections following PrP expression. *C*–*J*, representative images of the axonal projections for 1- (*C*–*F*) and 35-day-old (*G*–*J*) flies expressing LacZ or PrP constructs (*OK107-Gal4; CD8-GFP*). Control flies (*UAS-LacZ*) display robust axonal projection at days 1 (*C*) and 35 (*G*). Flies expressing PrP-WT (*UAS-human PrP-V129*) show thin, weak axonal projections at day 1 (*D*) that shrink by day 35 (*H*). Note the extensive blebbing. Flies expressing N174S (*UAS-human PrP-N174S*) display thin projections at day 1 (*E*) that do not shrink by day 35 (*I*). Flies expressing Y225A (*UAS-human PrP-Y225A*) display misshapen axonal projections at day 1 (*F*) that do not shrink by day 35 (*J*). *B* and *K*, scatter plots for axonal projection areas (*B*) and integrated intensity (*K*). Significant differences by pairwise t-tests and Holms *post hoc* analysis are illustrated. See Tables 2 and 3 for *p*-values. PrP, Prion protein.

We next examined the impact N174S and Y225A in quantitative assays in mushroom body neurons. The mushroom bodies are brain centers involved in higher neural processing in insects, including memory and learning (53, 54). Each mushroom body contains around 2000 neurons (Kenyon cells) clustered in the posterior brain with their dendrites projecting underneath the cell bodies (Fig. 8*A*). The axons project to the front of the brain, where the terminals branch into α, β, or γ projections (Fig. 8*A*). We constitutively expressed LacZ (control) or PrP in mushroom body neurons labeled by GFP (*OK107-Gal4; UAS-mCD8-GFP*) and imaged the brains in 1- and 35-day-old flies. First, we quantified total surface (Fig. 8*B*) and signal intensity (Fig. 8*K*) for the axonal projections. Two-way ANOVA showed significant effects of genotype and genotype-by-age for axonal area and of genotype and age for axonal intensity (Tables 2 and 3). Control 1-day-old flies expressing LacZ display robust axonal lobes (Fig. 8*C*) that are preserved in 35-day-old flies (Fig. 8*G*). 1-day-old flies expressing PrP-WT exhibit smaller axonal projections with lower intensity and membrane blebbing (Fig. 8*D*, arrows). 35-day-old flies exhibit prominent loss of projections and intense blebbing (Fig. 8*H*). 1-day-old flies expressing Y225A show better preservation of axonal projections than flies expressing PrP-WT but still thinner than control flies (Fig. 8, *E* and *F*). 35-day-old flies expressing N174S or Y225A (Fig. 8, *I* and *J*) show better preserved axonal terminals compared with older flies expressing WT. Overall, PrP-WT induces robust thinning of α, β, and γ projections and membrane blebbing, and the N174S and Y225A mutations are partially protective during aging.

**Table 2:** Summary of statistical analysis of the area of the axonal projections

a. Analysis of variance					
Source	DF	Sum of squares	Mean square	F ratio	Prob > F
Model	7	70,043,555	10,006,222	51.0420	
Error	96	18,819,747	196039.03		
C. Total	103	88,863,302			<0.0001^e^


b. Effect tests					
Source	parm^d^	DF	Sum of squares	F ratio	Prob > F
Genotype	3	3	62,456,335	106.1971	<0.0001^e^
Age (days)	1	1	4445	0.0227	0.8806
Age (days)∗Genotype	3	3	6,823,422	11.6021	<0.0001^e^


c. *Post hoc* Holm’s test for multiple comparisons					
Group 1	Group 2	Rank	TTest *p*-value	Holm’s *p*-value	Significant
D35, LacZ	D35, WT	1	0.0001	0.00172	Y
D35, LacZ	D35, N174S	5	0.0001	0.002	Y
D35, LacZ	D35, Y225A	6	0.0001	0.00208	Y
D1, LacZ	D1, WT	8	0.0001	0.00227	Y
D1, LacZ	D1, N174S	9	0.0001	0.00238	Y
D35, Y225A	D35, WT	11	0.0001	0.00263	Y
D35, N174S	D35, WT	12	0.0001	0.00277	Y
D1, LacZ	D1, Y225A	15	0.0001	0.00333	Y
D1, WT	D35, WT	17	0.0001	0.00384	Y
D1, Y225A	D1, WT	18	0.0002	0.00416	Y
D35, LacZ	D1, LacZ	21	0.0045	0.00555	Y
D1, Y225A	D1, N174S	22	0.0522	0.00625	N
D1, N174S	D1, WT	23	0.0724	0.00714	N
D35, N174S	D1, N174S	25	0.1745	0.01	N
D1, Y225A	D35, Y225A	27	0.6978	0.01666	N
D35, Y225A	D35, N174S	28	0.9539	0.025	N

Abbreviation: DF, degrees of freedom.

a, 2-way ANOVA shows significant differences. b, Effect tests by genotype, age, and genotype-by-age. c, Post hoc Holm’s test for multiple pairwise comparisons. Only relevant pairwise interactions are shown, but the rank order is preserved from all comparisons.

d parm: number of parameters.

e Significant differences.

**Table 3:** Summary of statistical analysis of the integrated density of the axonal projections

a. Analysis of variance					
Source	DF	Sum of squares	Mean square	F ratio	Prob > F
Model	7	1.5155e+14	2.165e+13	46.3978	
Error	95	4.4329e+13	4.666e+11		
C. Total	102	1.9588e+14			<0.0001^d^


b. Effect tests					
Source	parm^e^	DF	Sum of squares	F ratio	Prob > F
Genotype	3	3	1.0121e+14	72.3011	<0.0001^d^
Age (days)	1	1	1.9965e+13	42.7863	<0.0001^d^
Age (days)∗Genotype	3	3	9.436e+12	6.7407	0.0004^d^


c. *Post hoc* Holm’s test for multiple comparisons					
Subject 1	Subject 2	Rank	TTest *p*-value	Holm *p*-value	Significant
D35, LacZ	D35, WT	2	0.0001	0.00178	Y
D35, LacZ	D35, Y225A	4	0.0001	0.00192	Y
D1, LacZ	D1, WT	5	0.0001	0.002	Y
D35, LacZ	D35, N174S	8	0.0001	0.00227	Y
D1, N174S	D1, WT	11	0.0001	0.00263	Y
D1, N174S	D35, N174S	12	0.0001	0.00277	Y
D1, LacZ	D1, Y225A	13	0.0001	0.00294	Y
D1, Y225A	D35, Y225A	16	0.0001	0.00357	Y
D1, Y225A	D1, WT	17	0.0001	0.00384	Y
D1, N174S	D1, Y225A	19	0.0006	0.00454	Y
D1, LacZ	D1, N174S	20	0.0008	0.005	Y
D1, WT	D35, WT	21	0.0054	0.00555	Y
D35, N174S	D35, WT	22	0.0055	0.00625	Y
D35, Y225A	D35, WT	24	0.0104	0.00833	N
D35, N174S	D35, Y225A	25	0.786	0.01	N
D1, LacZ	D35, LacZ	28	0.9539	0.025	N

a, 2-way ANOVA shows significant differences. b, Effect tests by genotype, age, and genotype-by-age. c, Post hoc Holm’s test for multiple pairwise comparisons. Only relevant pairwise interactions are shown, but the rank order is preserved from all comparisons.

d Significant differences.

e parm: number of parameters.

**Figure 9:**
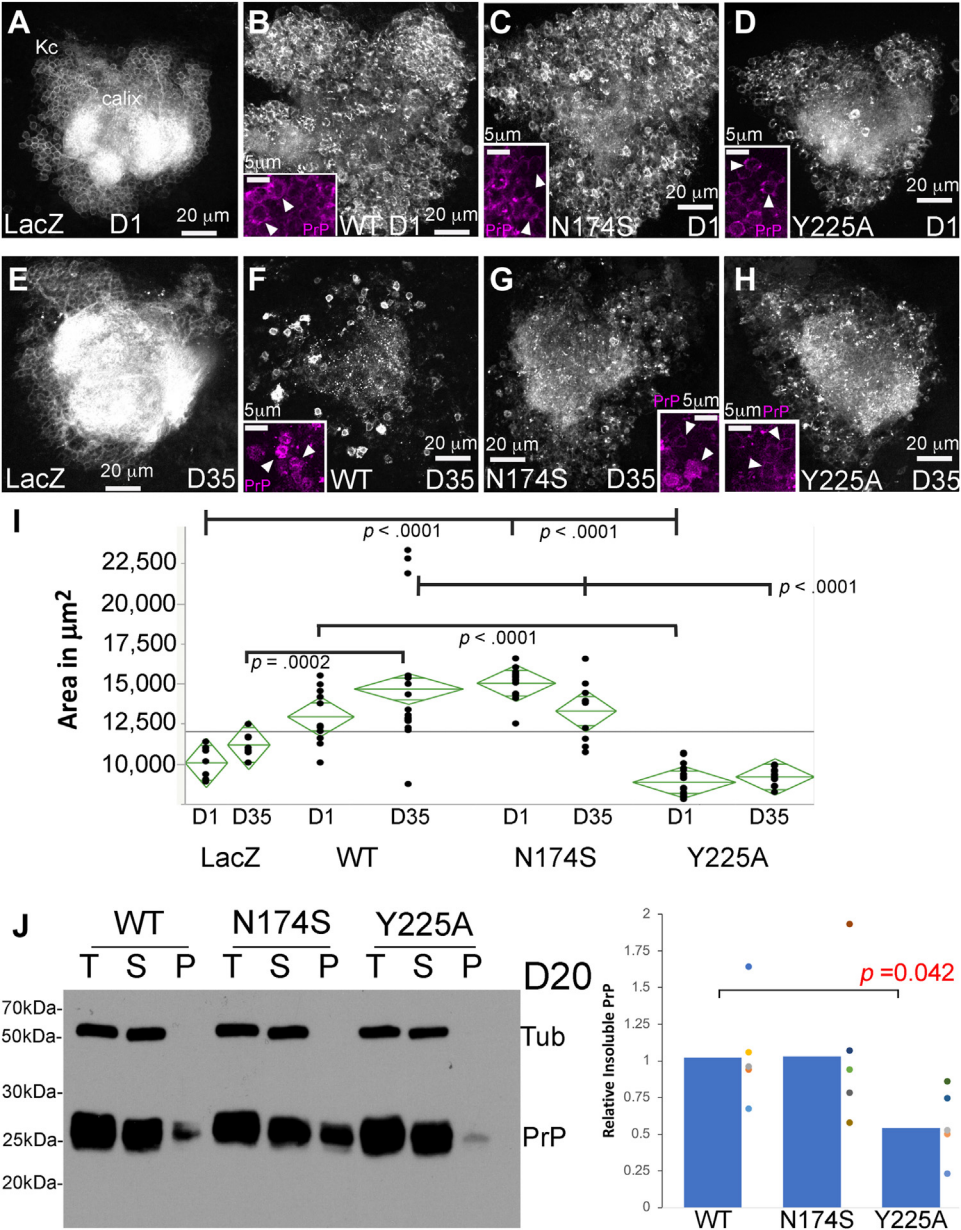
**N174S and Y225A lower the toxicity of human PrP in mushroom body cell bodies.***A*–*H*, genotypes are the same as in Figure 6. Some brains were immunostained with 3F4 anti-PrP antibody to examine PrP distribution (*insets*). Control flies expressing LacZ display compact, intense cell clusters at days 1 (*A*) and 35 (*E*). Flies expressing PrP-WT exhibit larger clusters at day 1 (*B*) that continue to expand by day 35 (*F*). Note the swollen cell bodies and the accumulation of intracellular PrP (*B* and *F*, insets). Flies expressing N174S display enlarged clusters at day 1 (*C*) that shrink by day 35 (*G*). Some cell bodies appear swollen and accumulate intracellular PrP (*C* and *G*, *insets*). Flies expressing Y225A display small clusters at day 1 (*D*) that do not shrink by day 35 (*H*). The cell bodies are small and do not progressively accumulate intracellular PrP (*D* and *H*, *insets*). *I*, scatter plot for the area of cell body clusters for 1- and 35-day-old flies. Significant differences by pairwise t-tests and Holms *post hoc* analysis are illustrated. See Table 4 for *p*-values. *J*, NaPTA insolubility assay. Flies were aged for 20 days and heads homogenized. The homogenates were incubated in sarkosyl/NaPTA. An aliquot was used as control (T-total), and the rest was centrifuged to separate the soluble (S) and insoluble (P-pellet) fractions. The experiment was repeated in five biological replicates and quantified. PrP-WT and N174S accumulated similar amounts of insoluble PrP, whereas Y225A accumulated about half. NaPTA, Na+ phosphotungstic acid; PrP, Prion protein.

We next examined the effects on Kenyon cell area (Fig. 9). Two-way ANOVA showed significant effects of genotype and genotype-by-age (Table 4). The Kenyon cell clusters experience no significant changes during aging in control flies expressing LacZ (Fig. 9, *A*, *E* and *I*). Surprisingly, 1-day-old flies expressing WT show enlarged clusters that continue to expand with aging while they also experience lower density indicative of cell loss (Fig. 9, *B* and *F* and Table 4). The cell bodies appear swollen and accumulate increasing amounts of PrP as they age (Fig. 9, *B* and *F*, insets). This phenotype is consistent with aberrant autophagy described previously in flies expressing the amyloid-β peptide (55, 56). 1- and 35-day-old flies expressing N174S show a similar expansion and intracellular PrP accumulation as that of flies expressing PrP-WT (Fig. 9, *C*, *G* and *I* and Table 4). In contrast, 1- and 35-day-old flies expressing Y225A have significantly smaller clusters than flies expressing PrP-WT (Fig. 9, *D*, *H*, and *I*) and accumulate low amounts of PrP compared to flies expressing WT (Fig. 9, *D* and *H*, insets). The lack of significant effect by age alone seems to stem from the different effects observed on the clusters by genotypes: enlargement, shrinking, or no change.

**Table 4:** Summary of statistical analysis of the area of the Kenyon cell clusters

a. Analysis of variance					
Source	DF	Sum of squares	Mean square	F ratio	Prob > F
Model	7	575,614,234	82,230,605	19.6491	
Error	88	368,276,203	4184956.9		
C. Total	95	943,890,438			<0.0001^d^


b. Effect tests					
Source	parm^e^	DF	Sum of squares	F ratio	Prob > F
Genotype	3	3	481,137,511	38.3228	<0.0001^d^
Age (Days)	1	1	2,815,517	0.6728	0.4143^d^
Age (Days)∗Genotype	3	3	39,849,191	3.1740	0.0281^d^


c. *Post hoc* Holm’s test for multiple comparisons					
Subject 1	Subject 2	Rank	TTest *p*-value	Holm *p*-value	Significant
D1, N174S	D1, Y225A	1	0.0001	0.00172	Y
D35, WT	D35, Y225A	4	0.0001	0.00192	Y
D1, N174S	D1, LacZ	5	0.0001	0.002	Y
D35, N174S	D35, Y225A	8	0.0001	0.00227	Y
D1, WT	D1, Y225A	9	0.0001	0.00238	Y
D35, WT	D35, LacZ	12	0.0002	0.00277	Y
D1, WT	D1, LacZ	14	0.0047	0.00312	N
D1, N174S	D1, WT	16	0.0142	0.00357	N
D35, WT	D1, WT	17	0.0293	0.00384	N
D35, N174S	D35, LacZ	18	0.0393	0.00416	N
D35, LacZ	D35, Y225A	19	0.0408	0.00454	N
D1, N174S	D35, N174S	20	0.0462	0.005	N
D35, WT	D35, N174S	22	0.0913	0.00625	N
D1, LacZ	D1, Y225A	23	0.1918	0.00714	N
D35, LacZ	D1, LacZ	24	0.3092	0.00833	N
D35, Y225A	D1, Y225A	27	0.6574	0.01666	N

a, 2-way ANOVA shows significant differences. b, Effect tests by genotype, age, and genotype-by-age. c, Post hoc Holm’s test for multiple pairwise comparisons. Only relevant pairwise interactions are shown, but the rank order is preserved from all comparisons.

d Significant differences.

e parm: number of parameters.

Last, analysis of the surface of the dendritic fields showed significant effects of genotype and genotype-by-age, but not age alone (Table S3). 1-day-old flies expressing LacZ display compact calyces that expand slightly in 35-day-old flies (Fig. S3, *A* and *E*). The calyx is organized in microglomeruli, in which boutons from ascending neurons are surrounded by dendritic claws, giving the calix a punctate appearance. This architecture is preserved in 35-day-old control flies (Fig. S3, *L* and *P*). 1-day-old flies expressing PrP-WT have smaller and highly disorganized calyces, with no visible microglomeruli (Fig. S3, *B* and *F*). The calyces continue to shrink and lose density in 35-day-old flies (Fig. S3, *M* and *Q*). The calices in flies expressing N174S or Y225A have normal sizes at days 1 and 35 but appear disorganized (Fig. S3, *C*, *D*, *G*, *H*, *N*, *O*, *R*, and *S*). The only significant differences are between 35-day-old flies expressing PrP-WT and all others (Fig. S3*W*). Overall, these data show the high toxicity of PrP-WT in the dendritic terminals and support the protective activity of Y225A.

### Y225A decreases abundance of misfolded PrP

We last examined the accumulation of misfolded/insoluble PrP conformations by NaPTA precipitation assay. NaPTA is known to selectively bind and precipitate disease-relevant PrP conformations (57, 58, 59). We showed previously that hamster PrP expressed in flies accumulates in PrP conformations that precipitate with NaPTA (26). We expressed human PrP-WT, N174S, or Y225A and aged the flies for 20 days. Then, we incubated head homogenates with sarkosyl and NaPTA followed by centrifugation to precipitate insoluble complexes and PAGE. No tubulin is present in any of the insoluble fractions, indicating the lack of contamination of the pellet (P) (Fig. 7*J*). Similar amounts of PrP-WT and N174S appeared in the pellets, confirming the ability of human PrP to accumulate in NaPTA-insoluble conformations. However, Y225A accumulated around 50% less insoluble PrP, indicating a change in the normal dynamics of human PrP to misfold into PrP^Sc^-like conformations. This is consistent with the predictions from the MD simulations and the lower toxicity shown above.

## Discussion

Rabbits have long been recognized for their low susceptibility to prion transmission (9, 10, 60). Recent experiments showed that following *in vitro* adaptation of rabbit PrP, rabbits still show limited infectivity (25, 61). Structural, *in vitro*, and *ex vivo* studies identified the critical role of S174 in promoting the stability of rabbit PrP (24, 30, 31). S174 forms a helix-capping domain with N171 that stabilizes α-helix 2 along with long-range effects on the β2-α2 loop and α-helix 3 (30, 31). Pig is one of the few mammals with S174 and also shows low susceptibility to prion diseases, but it is unclear whether it forms the helix-capping domain (62). Yet, current evidence for the protective role of S174 is mixed. N174S decreases the replication of mouse PrP (24), while rabbit PrP-S174N shows increased mobility of its β2-α2 loop *in silico* and lower resistance to denaturation *in vitro* (30, 31). On the other hand, rabbit PrP-S174N does not exhibit increase *in vitro* replication (32) nor shows toxicity in transgenic flies (29). We show for the first time the impact of S174 in the context of human PrP and report mild protective effects of N174S in *Drosophila* eye and mushroom body assays likely due to a small impact on the highly dynamic human PrP.

We examined in more detail the impact of a new rabbit PrP-specific residue A225—on human PrP dynamics and toxicity. MD simulations revealed significant differences between human PrP-WT and Y225A. In WT, Y225 interacts with Y169 through aromatic interactions, allowing the β2-α2 loop to adopt several conformations connected by low-energy barriers. Clusters 2 to 6 are characterized by β-turns in which the hydrophobic side chain of Y169 is exposed to the solvent as described previously in mouse PrP (42), with the important difference that the 3_10_-helix is more stable in mouse PrP than in human PrP. The Y225A substitution eliminates the aromatic interaction with Y169, which shifts the loop almost exclusively to the 3_10_-helical turn. In this conformation, the side chain of Y169 forms a hydrogen bond with the side chain of D178 along with aromatic interactions with F175, as previously found for mouse PrP (41, 42). Populating the 3_10_-helix results in a less dynamic β2-α2 loop with less overall exposed hydrophobic surface, mostly attributed to the shielding of Y169 (42). This impacts PrP aggregation because solvent exposure of hydrophobic (aromatic) sites is associated with aggregation (63, 64, 65). Overall, the significant conformational changes and increased stability of Y225A predicts lower toxicity and aggregation in flies.

We recently generated new *Drosophila* models expressing human PrP-WT, showing high toxicity in eyes and brain neurons compared to mouse and hamster PrP (46). We showed previously that PrP from rabbit, dog, and horse are not toxic when expressed in flies (28, 29), These differences reveal the unique conformational dynamics of human PrP making it more prone to misfold and cause disease. We show here that *Drosophila*-expressed human PrP acquires less *N*-glycosylation with a lack of the complex sialylation compared to PrP from human brain homogenates. This has the potential to impact the stability and toxicity of PrP, although glycosylation has no impact on prion disease or transmission, only on strain preservation (66, 67). Yet, the different toxicity observed among diverse mammalian PrPs following expression under comparable conditions in flies suggests that the high toxicity of human PrP is intrinsic, that is, mediated by its unique sequence. Here, we leveraged the high toxicity of human PrP in flies to examine the ability of N174S or Y225A to lower its toxicity *in vivo*. Expression of PrP-Y225A shows significantly lower toxicity in the eye and mushroom body neurons than WT. In addition, Y225A shows no Kenyon cell cluster expansion as opposed to flies expressing WT and N174S. This is a distinctive phenotype that is observed in young flies indicating early cellular manifestations of PrP-WT toxicity. Moreover, human PrP-WT accumulates in NaPTA-insoluble conformations in flies indicative of PrP misfolding and aggregation into disease-relevant conformations (57, 59). Y225A significantly decreases the amounts of NaPTA-insoluble PrP, indicating lower misfolding and aggregation consistent with the higher conformational stability predicted *in silico*. Although we have not completed a thorough panel of biochemical and neurotoxicity assays, these findings are highly supportive of the protective effect of the Y225A substitution on the human PrP backbone.

Why is the effect of Y225A more robust than that of N174S in lowering human PrP toxicity? Even if N174S forms the helix-capping domain (30), Y225 can still promote the solvent exposure of Y169. In contrast, Y225A has long-range effects that stabilize the β2-α2 loop in a 3_10_-helix conformation. Additional data for the contribution of the C-terminus to the stability of the entire CT3DD is available from other species. The marsupial tammar wallaby carries A225-A226 compared to A225-Y226 in rabbit and Y225-Y226 in human and most mammals (68). Of these two tyrosines, only Y225 interacts with the loop and increases its conformational plasticity. No experimental data is available for the susceptibility of the wallaby to prion diseases, but its NMR structure reveal a well-defined 3_10_-helical turn in the loop likely due to A225-A226 (69). Moreover, the NMR structure for mouse PrP-Y225A shows an increase in the structural definition of the loop, which supports our results with human PrP-Y225A (68). Overall, Y225-Y226, particularly Y225, play a key role in the high conformational dynamics of the CT3DD in human PrP.

Despite the identification of two residues impacting human PrP dynamics and toxicity, N174S and Y225A still show moderate and mild toxicity, respectively. These results illustrate the advantages of using the fast and economical *Drosophila* model to conduct exploratory toxicity assays. The conformational changes induced by N174S or Y225A do not prevent its misfolding nor eliminate its toxicity, highlighting the extreme structural features of human PrP (46). The remaining toxicity in human PrP-N174S or Y225A indicates that other residues contribute to the instability of human PrP. These results dispel the notion that any single amino acid encodes the different susceptibilities of mammals to prion disease. S174 and A225 may work cooperatively to stabilize rabbit PrP since it has been shown that both these residues have long-range effects on the CT3DD. It is also possible that small changes in charge or size in other positions contribute to the stability of the CT3DD, suggesting that multiple small changes are required to stabilize human PrP. Our analysis identifies multiple residues that participate in hydrogen bonds and hydrophobic interactions to stabilize different conformations, which complicates the identification of the key residues and conformations encoding PrP toxicity. Identifying the rules governing PrP toxicity will require introducing double and triple mutations in the human PrP backbone and the combination of structural and *in vivo* studies that marries sequence, morphotype, and phenotype. Decoding the rules governing PrP misfolding and toxicity will close a significant gap in our understanding of the mechanism underlying prion disease pathology. Additionally, this will be a significant step for developing compounds that stabilize nontoxic conformations.

## Experimental procedures

### Sequence alignment and 3D protein visualization

The alignment of the globular domain of human and rabbit PrP sequences was done using ClustalW2 (www.ebi.ac.uk/Tools/clustalw2). We used human PrP as reference: amino acid numbering for both species refers to the corresponding amino acid in human PrP (see Fig. 1*A*). Amino acid sequences were obtained from NCBI with the following accession numbers: AAH22532 (human) and AAD01554 (rabbit). The color-coded amino acids indicate properties relevant for protein structure (polarity and charge). To generate 3D views of human and rabbit PrP, we opened in PyMOL v.2.3.0 (pymol.org) the NMR structures for human (1QM2) and rabbit (2FJ3) PrP deposited in the RSCB Protein Data Bank (rcsb.org/pdb). We displayed the proteins in Cartoon formats showing only relevant amino acids. The 3D alignment of human and rabbit PrP was done by merging the two structures in PyMOL.

### MD simulations

The starting structure for the human PrP-WT encompassing residues 125 to 228 was obtained from the Protein Data Bank, PDB (1QM2) (13), after which M129 was mutated to V129, and the ring of H187 was rotated 180° for a better hydrogen bond between the N_ε_H and the carbonyl of R156. The Y225A mutant was obtained by mutating the previously generated human PrP-WT. All mutations and structure manipulations were performed using PyMOL v.2.3.0. MD simulations were carried out with the GROMACS software version 2018.3 (http://ftp.gromacs.org/pub/gromacs/gromacs-2018.3.tar.gz) (70) using the CHARMM 36m force field (71, 72). His155 and His187 were set to neutral with proton on the N_ε_ based on the surrounding environment and their estimated pKa (6.16 and 5.18, respectively) from PROPKA version 3.4 (73). Using the same approach, histidine residues 140 and 177 were considered positively charged (estimated pKa were 7.34 and 7.46, respectively). All other residues and termini were set to their standard protonation state at pH 7. Both systems were solvated in a ∼375 nm^3^ rhombic dodecahedron box with 11,966 CHARMM-modified TIP3P water molecules (74, 75), to which 36 K^+^ and 35 Cl^−^ ions were added to achieve electroneutrality and an ionic strength of 150 mM. Simulations were carried out in the Minnesota Supercomputing Institute at the University of Minnesota.

With harmonic restraints of 1000 kJ mol^−1^ nm^−2^ on the position of the heavy atoms, the systems were minimized using the steepest descent method until the largest force was less than 1000 kJ mol^−1^ nm^−1^, followed by 1 ns of molecular dynamics in the NVT ensemble. Next, the harmonic restraints were gradually released over 3 ns of simulations in the NPT ensemble, followed by 100 ns of unrestrained dynamics. Dynamics were propagated using the Leap-Frog integrator (76) with a time step of 2 fs, and all bonds involving hydrogen were constrained with the LINCS algorithm (70). Independent temperature coupling was performed for the protein and the solvent at 310 K with the velocity-rescaling algorithm (77) and a decay time constant of 0.1 ps in the initial restrain-release phase and with the Nosé-Hoover algorithm (78, 79) and a decay time constant of 2 ps subsequently. Pressure coupling at 1 atm was achieved using the Berendsen algorithm (80), with a decay time constant of 2 ps in the initial restrain-release phase and with the Parrinello-Rahman barostat (81) and a decay time constant of 4 ps subsequently. The Lennard–Jones interactions were force-switched between 1.0 and 1.2 nm, and electrostatic interactions were treated with the particle mesh Ewald method (82) using a 0.12 nm grid spacing and a fourth-order spline interpolation.

All T-REMDs were initiated from the same last structure of the initial 100 ns simulation. The temperature ramp between 310 K and 360 K was generated with the online temperature generator (83), which resulted in 23 replicas. During the first 5 ns, the replicas were allowed to equilibrate to the new temperature, and no exchanges were attempted. After the initial equilibration, the simulations were propagated for 200 ns, with exchanges attempted every 1 ps. Exchange probabilities were confirmed to be between 20% and 26%. To remove possible bias toward the starting structure, we discarded the initial 100 ns of each T-REMD simulation and used only the last 100 ns of the 310 K simulations for analyses, with structures saved every 5 ps. For both WT and Y225A mutant, we then tested the reproducibility of our results by selecting 23 random structures from the last 100 ns of T-REMD simulations and used these as starting structures for another round of 100 ns T-REMD simulations by re-initializing the velocities. Because the results obtained from the initial 100 ns and from the second set of simulations were comparable, we merged the data for the 310K replica from both sets of simulations and performed the analyses on this joined set of data.

### MD simulations analysis

PCA was employed to characterize the conformational landscape of the β2-α2 loop after the T-REMD trajectories for the human PrP-WT and -Y225A were combined in a single file. The software GROMACS (70) was used to calculate the dihedral φ and ψ angles for residues 164 to 175 of the combined trajectory and to perform PCA of the angles in the sine/cosine space. The PCA data of each individual trajectory were then projected on the common PCA eigenvectors and further processed and plotted with the R (84) software version 3.6.0 (https://cran.utstat.utoronto.ca/bin/windows/base/old/3.6.0/). 3D free energy isocontours were generated by Boltzmann weighting the density after binning the trajectories along the first three PCA eigenvectors and smoothing with 3D kernel density estimate. Clusters of similar conformations were identified using the density-based spatial clustering of applications with noise (85) using the Euclidean distance in the principal components space as a metric and setting the minimum number of points and the ε neighborhood parameters to 20 and 0.1, respectively. For each cluster, the φ and ψ dihedral angles were displayed as isodensity contours in Ramachandran plots.

The SASA (86, 87) was calculated for WT and Y225A trajectories with GROMACS (70) using a probe sphere radius of 0.14 nm and decomposed into individual amino acids contribution. Atoms with absolute value of the charge less than 0.2 e were considered hydrophobic and all other atoms hydrophilic. The RMSF of the main chain (N, Cα, C, O) was calculated to probe the local dynamics of the protein. Alignment of the protein’s main chain was done iteratively (88) and converged within three iterations.

The secondary structure of the β2-α2 loop was analyzed using the DSSP algorithm version 2.0 (39) in combination with the GROMACS software (70). In addition, the trajectories were further analyzed with the program PROMOTIF (40) version 2.0 to identify the type of turn. Ramachandran plots were created with the program R (84) by applying a 2D kernel density estimate to the distributions of dihedral angles.

The hydrogen bond analysis was performed with the HBonds plugin version 1.2 of VMD (89) version 1.9.3 using a cutoff of 0.35 nm between heavy atoms and a deviation from linearity of 30°. The amino acid contact maps were generated using the Bio3D plugin version 2.4-1 (69) of R. The distance matrix between heavy atoms was calculated at each frame, and a contact between two atoms was assigned if their distance was less than 0.45 nm. Once averaged over the trajectory, the resulting occupancy for every pair of atoms ranged from 0 to 1. The contact map difference plots were obtained by subtracting two contact maps (notice that when taking the difference between WT and Y225A contact maps, the missing side chain atoms of A225 in the mutant were assigned a value of 0).

The helical content of α-helix 3 was calculated using Plumed (90) version 2.4.3. The ALPHARMSD collective variable was applied to residues 214 to 228 using a switching function with values of 0.08 nm for r_0_ and n = 8 and m = 12 exponents. Considering that six consecutive residues are needed for helical recognition, the possible helical content ranges from 0 to 10 for the 15 residues analyzed.

### Generation of transgenic flies and fly genetics

Flies carrying the human PrP-V129 (45, 46), N174S, or Y225A were codon-optimized for *Drosophila* expression and chemically synthesized (Integrated DNA Technologies) taking advantage of their small size (<1 kb). Assembled sequences were cloned between *XhoI* and *XbaI* sites onto the pJFRC7-20XUAS-IVS-mCD8:GFP *Drosophila* expression vector [Addgene #26220, (91)] after removing the mCD8:GFP transgene. The final constructs were sequenced to verify their integrity. The attP-based constructs were microinjected into *yw* embryos expressing φ-C31 integrase (91) at Rainbow Transgenics following standard procedures (92) to generate transgenic lines for each plasmid. Two independent strains were established for each construct since they are all inserted in the same locus.

The driver strains *GMR-Gal4* (retina, all eye cells) (Mathew Freeman, Univ. of Oxford), *OK107-Gal4* (mushroom bodies) (93), and the reporters *UAS-LacZ* (94), *UAS-CD8-GFP* (49), and *UAS-GFP- KDEL* (K. Irvine) were obtained from the Bloomington *Drosophila* Stock Center (fly.bio.indiana.edu). Fly stocks were maintained on standard *Drosophila* medium at 25 °C. For experiments, homozygous females for the *Gal4* strains were crossed with *UAS* males to generate progeny-expressing *PrP* in the desired tissue. Crosses were placed at 25 °C for 2 days, transferred to 27 °C until the progeny completed development, and adults were aged at 27 °C, unless otherwise indicated. All assays were performed using females, unless otherwise indicated.

### Characterization of eyes

We expressed all the constructs in the eye under the control of *GMR-Gal4* at 25 °C for 2 days; the progeny was raised at 28 °C, and we collected adult females at day 1. Fresh eyes were described for changes with respect to controls: N-no change, E-enhancer, S-suppressor. Descriptors included three categories each scored from 0 (no change) to 3 (robust change): eye size, organization, and pigmentation. Changes were assessed from large progenies (at least 15–20 females) in three biological replicates (46). Aggregated scores were analyzed by one-way ANOVA in JMP Pro 16 followed by pairwise comparisons by Tukey honest significance test. Images are collected from flies with representative phenotypes out of large progenies of more than ten flies. To image fresh eyes, we froze the flies at −20 °C for at least 24 h and collected z-stacks with a Leica Z16 APO using a 2× Plan-Apo objective. Flattened in-focus images were produced with the Montage Multifocus module of the Leica Application Software. For scanning electron microscopy, flies were serially dehydrated in ethanol, critically dried, and metal-coated for observation in a Jeol JSM-6490LV.

### *Drosophila* homogenates, protein biochemistry, and Western blot

Ten flies per genotype and time point were used for analysis. Fly heads were homogenized in 100 μl of RIPA buffer containing Complete protease inhibitors (Roche) using a motorized pestle and centrifuged for 1 min at 1000 rpm. Ten microliters of supernatant was mixed with loading buffer and resolved by SDS-PAGE in 12% Mini-Protean TGX precast gel (Bio-Rad) under reducing conditions and electroblotted into nitrocellulose membranes. Membranes were blocked in TBS-T containing 5% nonfat milk and probed against the primary antibodies: anti-PrP 8H4 (1:10,000, Millipore-Sigma), anti-PrP 3F4 (1:10,000, Millipore-Sigma, Lot# 3150381), anti-β-Tubulin (1:50,000, Thermo Fisher Scientific, Lot# QC216972). The secondary antibody was anti-Mouse-HRP (1:4000) (Jackson ImmunoResearch). Immunoreactive bands were visualized by enhanced chemiluminescence (ProSignal Dura ECL, Genesee). The protein biochemistry protocols are described in more detail in (95).

For deglycosylation assays, *Drosophila* homogenates (10%, w/v) were made in lysis buffer (150 mM NaCl, 5 mM EDTA, 10 mM Tris–HCl, 0.5% sodium deoxycholate, 0.5% Nonidet P-40, pH 7.4). Homogenates were denatured by boiling in SDS sample buffer (3% SDS, 2 mM EDTA, 10% glycerol, l50 mM Tris–HCl, pH 6.8) for 10 min and precipitated with five volumes pre-chilled methanol. The pellets were denatured and deglycosylated by incubating with recombinant PNGase F according to the manufacturer’s protocol (Roche Applied Science). Deglycosylated samples were mixed with an equal volume of SDS sample buffer and boiled for 10 min and loaded for Western blot analysis.

For NaPTA precipitation (57, 96), we homogenized 20 flies per condition in 35 μl of Conversion buffer and spun for 2 min at 20,000 rpm to remove debris. Then, we mixed 30 μl of homogenate with 30 μl of 8% sarkosyl, incubated at 37 °C for 30 min, added 0.8 μl of MgCl_2_ at 0.1 M and 3 U of Benzonase, and mixed at 37 °C. Then, we added 4.5 μl of NaPTA 4% and incubated at 37 °C for 45 min at 800 rpm. We collected 32 μl for total PrP fraction and centrifuged the rest at 20,000 rpm for 45 min at 4 °C. We collected 32 μl of the supernatant, resuspended the pellet in 40 μl of SDS sample buffer (1×), resuspended the supernatant and total protein fractions each in 8 μl SDS sample buffer (5×), and resolved them together by PAGE. The membrane was incubated with Tubulin and PrP to ensure the specificity of the insoluble fraction containing no Tub.

### 2D Western blotting of PrP

2D Western blots were performed as previously described (52). In brief, tissue homogenates were boiled in SDS sample buffer (3% SDS, 2 mM EDTA, 4% β-mercaptoethanol, 10% glycerol, 50 mM Tris, pH 6.8), followed by precipitation by five volumes of prechilled methanol at −20 °C for 2 h and centrifugation at 14,000 rpm for 30 min at 4 °C. The pellets were resuspended in 50 μl reducing buffer (8 M urea, 2% CHAPS, 5 mM tributylphosphine, 20 mM Tris, pH 8.0) for 1 h at room temperature (RT) and added 5 μl iodoacetamide (200 mM) in dark at RT for greater than 1 h. Five volumes of prechilled methanol was added and incubated at −20 °C for 2 h and centrifuged at 14,000 rpm for 30 min at 4 °C. The pellets were resuspended in 200 μl of rehydration buffer (7 M urea, 2 M Thio-urea, 1% DTT, 1% CHAPS, 1% Triton X-100, 1% ampholyte pH 3–10, trace amount bromophenol blue) and centrifuged at 5000 rpm for 5 min at RT. The samples were loaded onto the immobilized pH gradient strips for rehydration at RT for more than 12 h with gentle shaking. The first isoelectric focusing was performed on the rehydrated gel strips for 7 h using a focusing tray. For the SDS-PAGE, the focused gel strips were equilibrated for 15 min each in equilibration buffer A (6 M urea, 2% SDS, 20% glycerol, 130 mM DTT, 0.375 M Tris–HCl, pH 8.8) and equilibration buffer B (6 M urea, 2% SDS, 20% glycerol, 135 mM iodoacetamide, 0.375 M Tris–HCl, pH 8.8), respectively. The equilibrated strips were loaded onto 15% Bio-Rad Criterion gels at 150 V for 90 min. The proteins in the gels were transferred to Immobilon-P membrane for 90 min at 0.35 A. The membranes were incubated in 5% milk for 1 h for blocking and incubated with primary antibody 3F4 (1:30,000) at RT for 2 h. Following incubation with the secondary antibody sheep anti-mouse IgG (1:3000) for 1 h, the PrP spots were visualized on Kodak films by ECL Plus as recommended by the manufacturer.

### Immunofluorescence, microscopy, and image analysis

For PrP immunofluorescence, we co-expressed the *PrP* constructs and the membrane marker m*CD8-GFP* under the control of *OK107-Gal4* (*UAS-CD8-GFP; OK107-Gal4/UAS-PrP*). Whole-mount immunofluorescence of fixed and permeabilized (0.3% Triton X-100) brains was conducted by incubating with the primary antibody (3F4 anti-PrP, 1: 1000) followed by the secondary anti-mouse-Cy3 antibody (Thermo Fisher Scientific) at 1:600 as described previously (28). For subcellular localization of PrP, we imaged single interneurons of the larval ventral ganglion using a 63× NA: 1.4 (oil) objective (Fig. 6) or adult mushroom body clusters using a 40× NA: 1.0 (water) objective (Figs. 8, 9 and S3).

For mushroom body degeneration, we crossed *OK107-Gal4; mCD8-GFP* flies with *LacZ* (negative control) or *PrP* constructs at 27 °C. *mCD8-GFP* is a robust chimeric membrane-bound GFP (97). Adult flies at day 1 post eclosion followed by aging for 35 days were fixed and mounted in Vectashield antifade (Vector) mounting medium for microscopic documentation of different regions of the mushroom body complexes—cell bodies (Kenyon cells), axonal lobes, and dendritic fields (calyx). To quantify the surface of the mushroom body structures, we collected Z-stacks in an LSM 710 Zeiss confocal system using a 40× objective NA: 1.0 (water). All samples were imaged with the same settings. Image processing: We flattened the Z-stacks (maximum intensity projections) and manually traced the regions of interest in Adobe Photoshop 2021 to calculate area or intensity from 15 to 20 independent images. Raw images were combined using Photoshop; processing included trimming of noninformative edges and brightness/contrast adjustment to whole images.

### Statistics

For Western blots directly from homogenates or following NaPTA precipitation, at least three independent experiments were completed. Films were scanned at high resolution, and the bands for Tubulin (loading control) and PrP were selected and quantified in Photoshop. Following normalization, relative PrP levels were averaged and pairwise T-tests were performed.

Mushroom body data (area or integrated intensity) were exported to JMP Pro 16 to calculate averages, SDs, create graphs, and conduct two-way ANOVA of the effects of genotype and age. Following the identification of differences in at least one variable, we calculated pairwise *t* test analyses for all pairs followed by *post hoc* correction using the Holm’s method for multiple comparisons (98) to identify significant differences. ANOVA descriptive analyses and relevant pairwise comparisons are shown in tables.

## Data availability

### Sharing model organisms

All the new PrP transgenic lines will be freely distributed to investigators at academic institutions interested in these flies for noncommercial research. The recipient investigators must provide written assurance and evidence that that these flies will not be further distributed by the recipient without the author’s consent and that these flies will not be used for commercial purposes. Requests from for-profit corporations to use these flies commercially will be negotiated by UMN technology transfer office.

### DNA constructs

Constructs and their sequences will be freely distributed upon request to academic investigators for nonprofit research.

### MD simulations

Files containing the trajectories of the MD simulations will be made available for validation and further analysis by depositing them in appropriate academic servers.

## Supporting information

This article contains supporting information.

## Conflict of interest

The authors declare that they have no conflicts of interest with the contents of this article.
