# Supplementary material for: Y225A induces long-range conformational changes in human prion protein that are protective in Drosophila
Source: J Biol Chem. 2023 Jun 2;299(7):104881. doi: 10.1016/j.jbc.2023.104881 (PMC10339063; doi:10.1016/j.jbc.2023.104881)
Supplement: Supplemental Figures S1–S3 [file mmc1.pdf]

Supplementary Materials

Supplementary Figure S1. Principal component analysis of eigenvalues and eigenvectors. a, Dihedral principal component analysis eigenvalues. The eigenvalues corresponding to the first three eigenvectors – used in the analysis and in figure 2 – are responsible for the largest variance as indicated. A dashed line shows the total variance divided by the 48 eigenvectors. **b**, dihedral principal component analysis eigenvectors. The first three eigenvectors are shown as a function of residue number. There are 4 elements per residue (two φ / ψ angles, and a sine a cosine for each angle).

Supplementary Figure S2. Coomassie staining of proteins subjected to 2D-PAGE from *Drosophila* expressing three human PrP variations. Panels **a** and **b**: proteins on 2D gel from *Drosophila* expressing human PrP-129V; **c** and **d**: PrP-N174S; **e** and **f**: PrP-Y225A. Panels **a**, **c**, and **e:** untreated samples (-) and **b**, **d**, and **f**: treated samples (+) with PNGase F for deglycosylation. All panels show Coomassie staining to confirm that the *Drosophila* samples achieve protein separation along the pI gradient. Same genotypes as in figure 7.

Supplementary Figure S3. N174S and Y225A ameliorate the toxicity of human PrP in mushroom body dendritic fields. a-v, Representative images of the calices for 1- (a-k) and 35-day-old (l-v) flies expressing LacZ or PrP in the mushroom bodies (*OK107-Gal4; CD8-GFP*). Quantification of the dendritic clusters was done by imaging CD8-GFP. Some brains were immuno-stained with 3F4 anti-PrP antibody to examine PrP distribution. Control flies expressing LacZ (*OK107-Gal4; CD8-GFP; UAS-LacZ*) display compact calices at days 1 (a and e) and 35 (l and p). Note the organization of the calix in microglomeruli. Flies expressing PrP-WT (*OK107-Gal4; CD8-GFP; UAS-human PrP-V129*) exhibit hypointense, disorganized calices at day 1 (b and f) that continue to degenerate by day 35 (o and s). Flies expressing N174S (*OK107-Gal4; CD8-GFP; UAS-human PrP-N174S*) or Y225A display hypointense, disorganized calices at day 1 (c, d, g, and h) that do not shrink by day 35 (n, o, r, and s). PrP shows a diffuse distribution in PrP-WT, N174S and Y225A at day 1 (i-k). By day 35, the intensity of PrP-WT is weak correlating with

the loss of GFP (t) whereas N174S and Y225A preserve the intensity and distribution (u and v). **w,** Scatter plot for the area of calyces for 1- and 35-day-old flies. Significant differences by pairwise t-tests and Holms *post hoc* analysis are illustrated. See Tables S2 for *p*-values.

Supplementary Materials

Figure S1

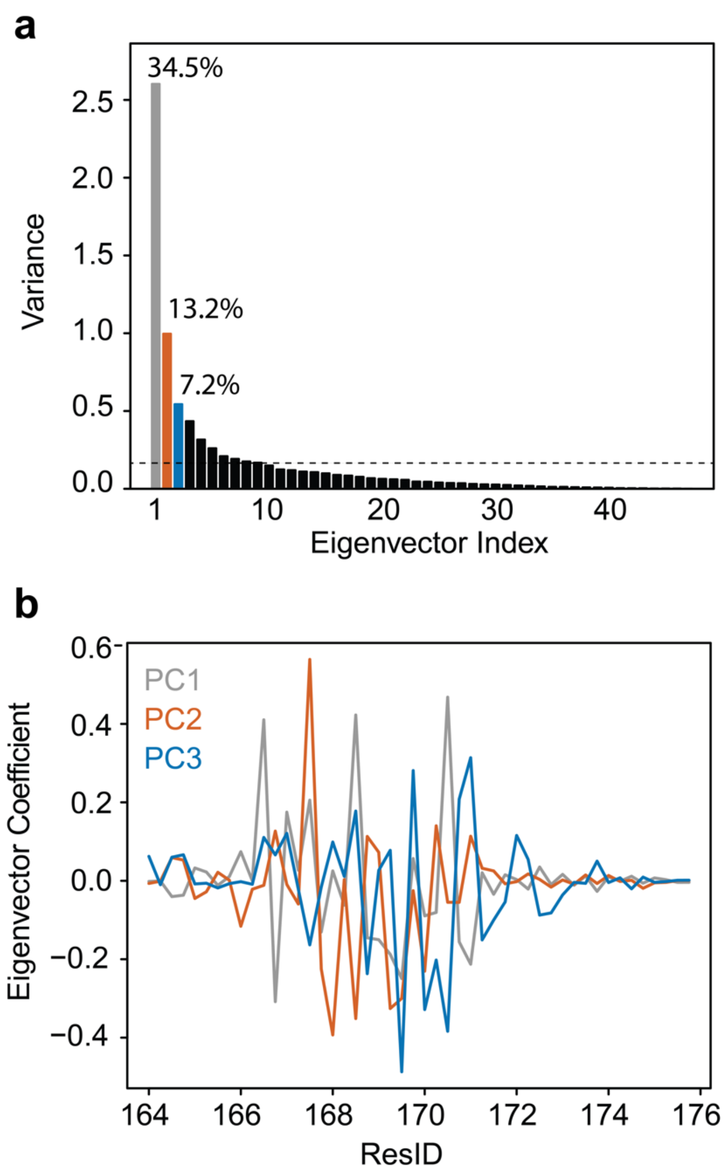

Figure S2

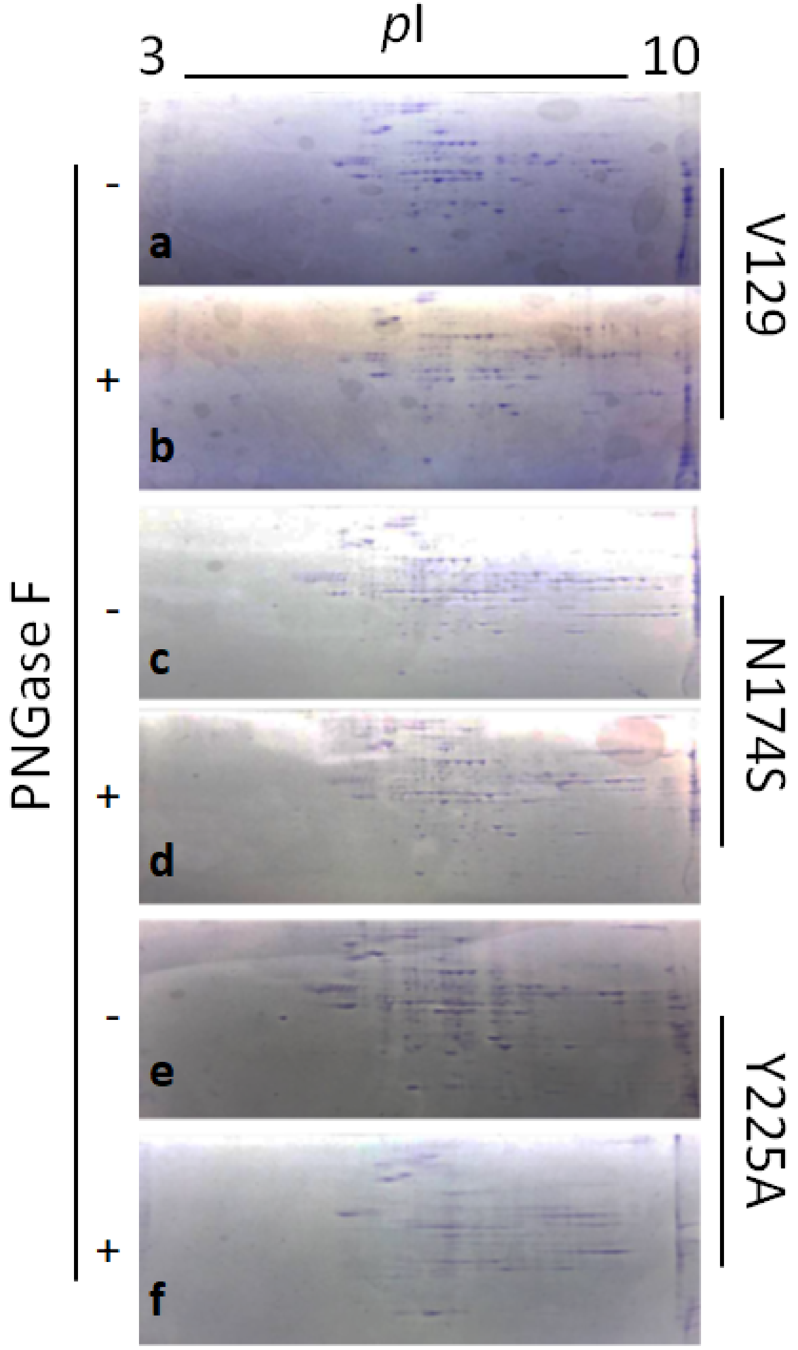

Figure S3

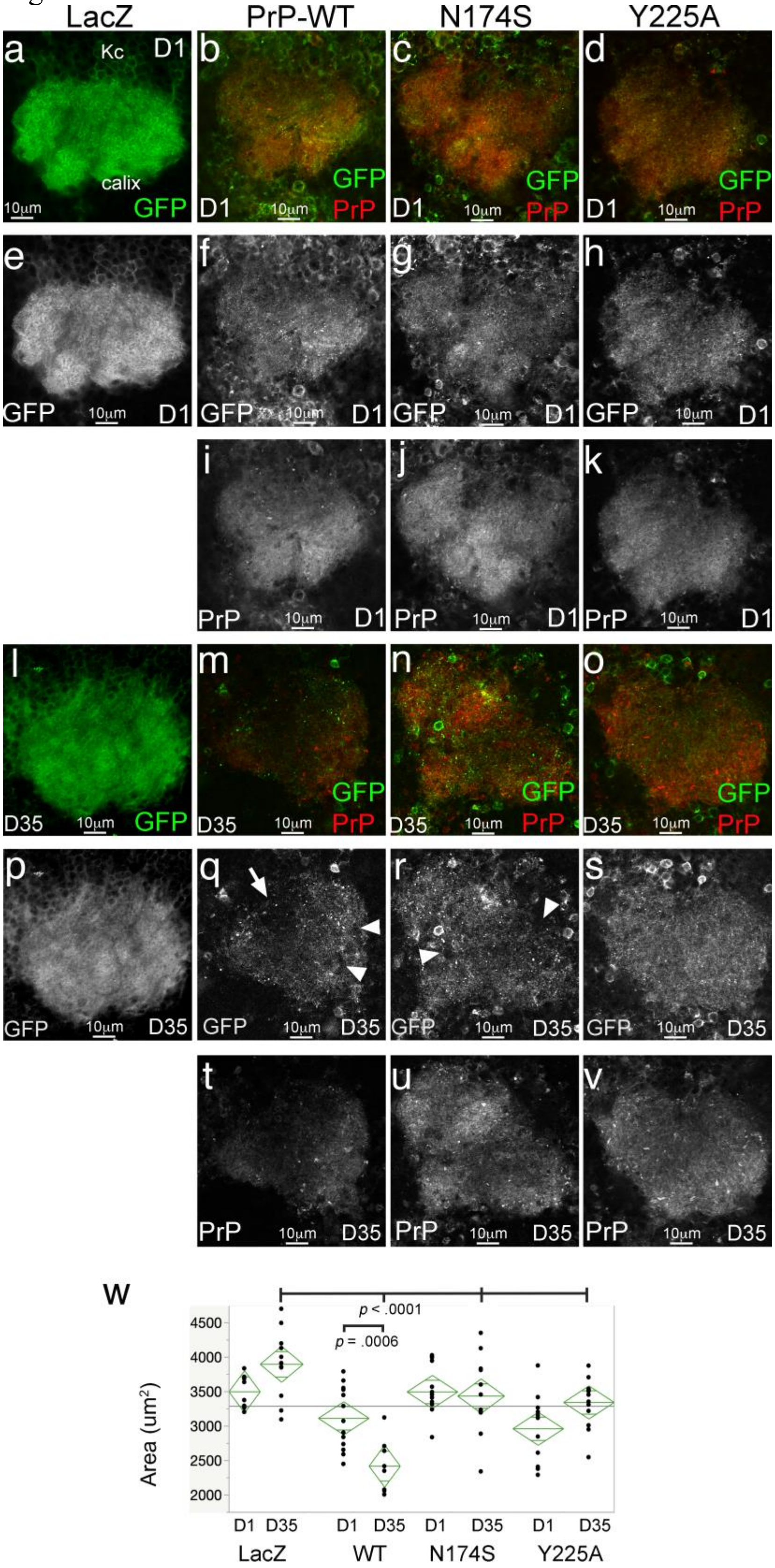
LacZ
PrP-WT
N174S
Y225A
a
Kc
D1
calix
10µm
GFP
b
c
d
GFP
PrP
D1
10µm
e
f
g
h
i
j
k
PrP
l
m
n
o
D35
p
q
r
s
t
u
v
w
p < .0001
p = .0006
Area (um²)
4500
4000
3500
3000
2500
2000
D1
D35
LacZ
WT
N174S
Y225A
