# Supplementary material for: Y225A induces long-range conformational changes in human prion protein that are protective in Drosophila
Source: J Biol Chem. 2023 Jun 2;299(7):104881. doi: 10.1016/j.jbc.2023.104881 (PMC10339063; doi:10.1016/j.jbc.2023.104881)
Supplement: Supplemental Tables S1–S3 [file mmc2.docx]

**Supplementary Tables**

**Supplementary Table S1. Hydrogen bonds detailed analysis.** Hydrogen bonds formed by the β2-α2 loop residues (163-175) and Y/A225 are listed, with “M” indicating main-chain, and “S” side chain. The percent occupancies are reported for every cluster, for both WT and Y225A. The cells are color coded to aid in distinguishing the differences: > 75% (light gray, bolded), 75% - 50% (underlined), 50% - 25% (italics), white < 25%. Dark gray cells have high occupancy (> 75%) for all clusters.

|  |  | **Wild Type** | | | | | | **Y225A** | | | | | |
| --- | --- | --- | --- | --- | --- | --- | --- | --- | --- | --- | --- | --- | --- |
| **Donor** | **Acceptor** | Cl.1 | Cl.2 | Cl.3 | Cl.4 | Cl.5 | Cl.6 | Cl.1 | Cl.2 | Cl.3 | Cl.4 | Cl.5 | Cl.6 |
| L125-M | E168-S | *44* | 2 | 0 | 3 | 0 | 0 | *41* | 1 | 0 | 0 | 0 | 0 |
| V129-M | Y163-M | 86 | 85 | 86 | 84 | 84 | 84 | 88 | 89 | 81 | 86 | 85 | 92 |
| Y163-M | V129-M | 81 | 81 | 82 | 82 | 83 | 80 | 80 | 87 | 77 | 82 | 79 | 75 |
| Y163-S | E221-S | 54 | 58 | 72 | 66 | **78** | *33* | 51 | *48* | 63 | 62 | 72 | 52 |
| R164-S | G126-M | 23 | *29* | 10 | 22 | 0 | 9 | *36* | 23 | 14 | 8 | 7 | 51 |
| R164-S | G127-M | *31* | 9 | 19 | 8 | 8 | *29* | 19 | 11 | 1 | *39* | 3 | 8 |
| R164-S | P165-M | 0 | 5 | 13 | 12 | **78** | 13 | 0 | 4 | 11 | 8 | 59 | 0 |
| R164-S | D167-S | 0 | 7 | *36* | 11 | **78** | *31* | 0 | 2 | **94** | 10 | 64 | 0 |
| R164-S | E168-S | *37* | 0 | 0 | 0 | 0 | 0 | 23 | 0 | 0 | 0 | 0 | 0 |
| R164-S | D178-S | 9 | *41* | *42* | 65 | **96** | *40* | 3 | 52 | **85** | *49* | **95** | 20 |
| M166-M | E221-S | 8 | *28* | 3 | 9 | 2 | 0 | 2 | 1 | 3 | 1 | 1 | 2 |
| E168-M | P165-M | *31* | 0 | 0 | 0 | 0 | 0 | *31* | 0 | 0 | 0 | 0 | 0 |
| Y169-M | M166-M | 65 | 0 | 0 | 0 | 0 | 0 | 66 | 0 | 0 | 0 | 0 | 0 |
| Y169-S | D178-S | **82** | 0 | 0 | 0 | 0 | 0 | **95** | 0 | 0 | 0 | 0 | 0 |
| Y169-S | S222-S | 0 | *37* | 0 | 15 | 0 | 0 | 0 | 5 | 0 | 4 | 0 | 0 |
| S170-M | A224-M | 0 | 0 | 0 | 0 | 0 | *35* | 0 | 0 | 0 | 0 | 0 | 0 |
| Q172-M | Q172-S | 13 | 1 | 4 | 4 | 15 | 7 | 6 | 4 | 2 | 6 | *30* | 0 |
| N173-M | N171-S | 11 | 3 | 5 | 13 | *45* | 4 | 13 | 5 | 8 | 14 | 56 | 2 |
| N174-M | N171-S | 54 | *33* | *49* | *28* | 3 | 55 | *47* | 21 | *33* | *36* | 12 | 52 |
| N174-S | E168-S | 0 | 0 | 0 | 4 | *47* | 0 | 0 | 0 | 0 | 4 | *41* | 0 |
| F175-M | N171-M | 61 | 73 | 74 | *48* | 1 | **76** | 54 | 73 | 60 | *44* | 10 | **77** |
| V176-M | Q172-M | 92 | 92 | 91 | 93 | 94 | 85 | 91 | 93 | 91 | 95 | 94 | 95 |
| H177-M | N173-M | 89 | 81 | 82 | 89 | 85 | 72 | 87 | 83 | 87 | 87 | 88 | 86 |
| D178-M | N174-M | 85 | 70 | 80 | 71 | 90 | 80 | 81 | 73 | 76 | 73 | 86 | 72 |
| C179-M | F175-M | 83 | 83 | 85 | 81 | 81 | 84 | 84 | 89 | 87 | 86 | 80 | 83 |
| Y218-S | E168-M | 0 | 0 | 0 | 7 | 69 | 0 | 0 | 0 | 0 | 6 | 53 | 0 |
| Y218-S | Y169-M | 1 | 0 | 0 | 53 | 3 | 0 | 0 | 0 | 0 | *49* | 13 | 0 |
| Y218-S | S170-M | 2 | **82** | *38* | 14 | 0 | 55 | 0 | *48* | *31* | 21 | 0 | 6 |
| Y218-S | S170-S | 0 | 0 | 16 | 0 | 0 | 19 | 0 | 0 | *27* | 0 | 0 | *45* |
| Y/A225-M | E221-M | 14 | 51 | 23 | *37* | *47* | 8 | 11 | 3 | *36* | 3 | 6 | 5 |
| R228-M | Y/A225-M | 2 | 12 | 7 | 1 | 10 | 5 | 3 | 1 | *28* | 1 | 1 | 3 |

**Supplementary Table S2. Summary of statistical analysis of the area of the dendritic fields.**

**a. Analysis of Variance**

**Source DF Sum of Squares Mean Square F Ratio Prob > F**

Model 7 13124592 1874942 9.9012

Error 83 15717352 189366

C. Total 90 28841943 <.0001*

**b. Effect Tests**

**Source # parm DF Sum of Squares F Ratio Prob > F**

Genotype 3 3 9735053.3 17.1363 <.0001*

Age (Days) 1 1 950.3 0.0050 0.9437

Age (Days)*Genotype 3 3 408277 2.2 7.1868 0.0002*

**c. *Post hoc* Holm’s test for multiple comparisons**

| **Subject 1** | **Subject 2** | **Rank** | **TTest P-value** | **Holm p-value** | **Significant** |
| --- | --- | --- | --- | --- | --- |
| D35, LacZ | D35, WT | 1 | 0.0001 | 0.00172 | **Y** |
| D35, N174S | D35, WT | 4 | 0.0001 | 0.00192 | **Y** |
| D35, Y225A | D35, WT | 6 | 0.0001 | 0.00208 | **Y** |
| D1, WT | D35, WT | 8 | 0.0006 | 0.00227 | **Y** |
| D1, N174S | D1, Y225A | 9 | 0.0024 | 0.00238 | N |
| D35, LacZ | D35, Y225A | 10 | 0.0026 | 0.0025 | N |
| D1, LacZ | D1, Y225A | 12 | 0.0074 | 0.00277 | N |
| D35, LacZ | D35, N174S | 14 | 0.0129 | 0.00312 | N |
| D1, N174S | D1, WT | 16 | 0.0276 | 0.00357 | N |
| D35, Y225A | D1, Y225A | 17 | 0.028 | 0.00384 | N |
| D35, LacZ | D1, LacZ | 18 | 0.0517 | 0.00416 | N |
| D1, LacZ | D1, WT | 19 | 0.0523 | 0.00454 | N |
| D1, WT | D1, Y225A | 23 | 0.377 | 0.00714 | N |
| D35, N174S | D35, Y225A | 25 | 0.5984 | 0.01 | N |
| D1, N174S | D35, N174S | 26 | 0.7296 | 0.0125 | N |
| D1, LacZ | D1, N174S | 28 | 0.9908 | 0.025 | N |

**a,** 2-way ANOVA shows significant differences. **b,** Effect tests by genotype, age, and genotype-by-age. **c,** *Post hoc* Holm’s test for multiple pairwise comparisons. Only relevant pairwise interactions are shown (significant in red), but the rank order is preserved from all comparisons. Same legend as in Table 2. *Significant differences.

**Supplementary Table S3. Analysis of Variance: Eye phenotype by Genotype**

1. **Source DF Sum of Squares Mean Square F Ratio**

Model 3 145.26075 48.4203 164.0645

Error 73 21.54444 0.2951 **Prob > F**

C. Total 76 166.80519 <.0001*

1. **Effect Tests**

**Source # parm DF Sum of Squares F Ratio Prob > F**

Genotype 3 3 145.26075 164.0645 <.0001*

1. **Least squares Means Differences Tukey honest significance:**

**Level- Level Difference Std Err Dif p-Value**

V129 GFP 3.500000 0.1717933 <.0001*

V129 Y225A 2.650000 0.1740391 <.0001*

N174S GFP 2.405556 0.1765008 <.0001*

N174S Y225A 1.555556 0.1786874 <.0001*

V129 N174S 1.094444 0.1765008 <.0001*

Y225A GFP 0.850000 0.1740391 <.0001*

DF: degrees of freedom, # parm: number of parameters

**a,** 1-way ANOVA shows significant differences. **b,** Effect tests by genotype. **c,** Tukey *Post hoc* analysis of significance for multiple pairwise comparisons. Significant pairwise differences are shown in red). Same legend as in Table S2. *Significant differences.
